# High-throughput sequencing unravels the cell heterogeneity of cerebrospinal fluid in the bacterial meningitis of children

**DOI:** 10.3389/fimmu.2022.872832

**Published:** 2022-09-02

**Authors:** Haihan Xiao, Haijuan Xiao, Yun Zhang, Lingyun Guo, Zhenzhen Dou, Linlin Liu, Liang Zhu, Wenya Feng, Bing Liu, Bing Hu, Tianming Chen, Gang Liu, Tingyi Wen

**Affiliations:** ^1^ CAS Key Laboratory of Pathogenic Microbiology and Immunology, Institute of Microbiology, Chinese Academy of Sciences, Beijing, China; ^2^ University of Chinese Academy of Sciences, Beijing, China; ^3^ Department of Infectious Diseases, Key Laboratory of Major Diseases in Children, Ministry of Education, Beijing Children’s Hospital, Capital Medical University, National Center for Children’s Health, Beijing, China; ^4^ Savaid Medical School, University of Chinese Academy of Sciences, Beijing, China

**Keywords:** bacterial meningitis, cerebrospinal fluid - CSF, cell heterogeneity, neuroinflammation, single-cell RNA sequencing (scRNA-seq)

## Abstract

Bacterial meningitis (BM) is a common life-threatening infection in children that occurs in the central nervous system (CNS). The cytologic examination of cerebrospinal fluid (CSF) is a key parameter in the diagnosis of BM, but the heterogeneity of cells in the CSF has not been elucidated, which limits the current understanding of BM neuroinflammation. In this study, CSF samples were collected from a number of BM patients who were in different stages of disease progression. Single-cell RNA-sequencing (scRNA-seq), with additional bulk transcriptome sequencing, was conducted to decipher the characteristics of CSF cells in BM progression. A total of 18 immune cell clusters in CSF were identified, including two neutrophils, two monocytes, one macrophage, four myeloid dendritic cells, five T cells, one natural killer cell, one B cell, one plasmacytoid dendritic cell, and one plasma cell subtype. Their population profiles and dynamics in the initial onset, remission, and recovery stages during BM progression were also characterized, which showed decreased proportions of myeloid cells and increased proportions of lymphoid cells with disease progression. One novel neutrophil subtype, FFAR2^+^TNFAIP6^+^ neutrophils, and one novel monocyte subtype, THBS1^+^IL1B^+^ monocytes, were discovered, and their quantity changes positively correlated with the intensity of the inflammatory response in the CSF during BM. In addition, the CSF of BM patients with unsatisfactory therapeutic responses presented with different cell heterogeneity compared to the CSF of BM patients with satisfactory therapeutic responses, and their CSF featured altered intercellular communications and increased proportions of type II myeloid dendritic cells and plasmacytoid dendritic cells. Moreover, the bulk transcriptome profiles of autologous CSF cells and peripheral blood leukocytes of BM patients showed that the immune cells in these two physiological compartments exhibited distinct immune responses under different onset conditions. In particular, the CSF cells showed a high expression of macrophage characteristic genes and a low expression of platelet characteristic genes compared with peripheral blood leukocytes. Our study conducted an in-depth exploration of the characteristics of CSF cells in BM progression, which provided novel insights into immune cell engagement in acute CNS infection.

## Introduction

Bacterial meningitis (BM) is a common infectious disease occurring in the central nervous system (CNS) (1). Its incidence remains relatively high in preterm infants and neonates with documented sepsis (2, 3). Up to half of the survivors have poor prognosis and may develop seizures, cognitive deficiencies, or hearing and visual impairments (4). In addition, although BM is considered an acute infection and could be treated with timely antimicrobial therapy and clinical care (5–7), there has been an increasing number of BM patients that are refractory to treatment who may experience a long illness duration or relapse (8, 9). All these factors indicate that BM is a significant threat to the health of children and requires more attention.

CSF is a fluid mainly secreted by the choroid plexus and is found in the ventricles of the brain and in the cranial and subarachnoid spaces (10). As part of the CNS, the CSF composition and its changes can reflect pathological processes in the CNS, and therefore the CSF offers a unique insight into CNS disorders (11). Normal CSF contains few cells with no more than five mononuclear (MN) cells per microliter, which are mainly lymphocytes (12). CNS disorders such as infection, inflammation, degeneration, and tumors can result in CSF pleocytosis, and therefore a CSF cytologic examination is an important test in the diagnosis of CNS disease (13, 14). Routine clinical CSF examinations based on morphologic workup have provided valuable information on the cytologic characteristics of CSF cells (15–17), such as total cell count (TCC) and typical cell types present in the CSF, including polymorphonuclear (PMN) cells (neutrophil granulocytes) and MN cells (lymphocytes and monocytes), but these tests only show preliminary cell types, and the heterogeneity of these cells is not well explored. Some studies have characterized the phenotype of CSF cells by immunostaining combined with flow cytometric analysis (18–20). However, as only a small set of surface markers was selected, the results are low in resolution and may miss important cells.

Next-generation sequencing (NGS)-based single-cell RNA-sequencing (RNA-seq) technology has become a powerful tool for characterizing the heterogeneous properties of cellular communities (21). Compared with traditional bulk transcriptome sequencing (bulkRNA-seq), scRNA-seq enables the efficient determination of gene expression of an individual cell within biospecimens (22, 23), which can uncover distinct cell types, classify cell subpopulations, and identify rare cells. In recent years, several researchers have investigated the characteristics of CSF cells under normal or disease conditions using scRNA-seq (24–29)—for instance, the CSF cells of healthy adults were found to be dominated by T cells with a smaller number of monocytes and dendritic cells. Similar heterogeneous compositions of CSF cells were found in HIV-infected adults. In patients with inflammatory demyelinating disease such as multiple sclerosis (MS), relapsing–remitting MS (RRMS), and anti-myelin oligodendrocyte glycoprotein disorder, the major cell types in CSF with pleocytosis were CD4^+^ and CD8^+^ T cells, with other cell types such as γδ T cells, natural killer (NK) cells, monocytes (MOs), macrophages (MФs), myeloid dendritic cells (mDCs), plasmacytoid dendritic cells (pDCs), neutrophils (NEUs), and B cells also identified at relatively low proportions. In the CSF of patients with brain metastases (BrM), the abundant cell types were MФs, T cells, and NK cells, and the less abundant cell populations were DCs and NEUs.

BM is an acute CNS infectious process in which bacterial pathogens penetrate the blood–brain barrier into meningeal compartments, causing a secondary immune response and neuroinflammatory dysfunction (30). Compared with CNS autoimmune and neurodegenerative disorders in which MN cells are the main participants of meningeal immunity, BM presents a different neuroinflammatory patterns because CSF pleocytosis is accompanied by elevated PMN cells (31, 32). Unfortunately, unlike the extensive research on the biochemistry of CSF supernatant parameters (16, 33–35), such as glucose, total protein, and cytokine contents as well as their relationship to BM progression, the mining of heterogeneous information on CSF cells in BM has not been well studied, and therefore there is little knowledge of the participation of immune cells in CNS during BM progression. In this study, we grouped the collected CSF samples into different stages of disease progression through a combined analysis of their hospital-based cytologic information from routine examination reports and the disease durations of the donors. scRNA-seq was performed to characterize the cell composition of the CSF, aiming to identify cell types, cell population compositions, and changes in these during BM progression. On the basis of scRNA-seq data, we also explored the changes in the cell population compositions and cell–cell communication of CSF cells in BM patients with satisfactory and unsatisfactory therapeutic effects as well as the transcriptomic difference between autologous peripheral blood leukocytes (PBLs) and CSF cells during BM development, with bulkRNA-seq providing additional data. We hope that our work will provide valuable insights for understanding the engagement of immune cells in the CNS during BM progression.

## Materials and methods

### Sample processing

The study was approved by the Ethics Committee of Beijing Children’s Hospital, Capital Medical University, and performed according to the Declaration of Helsinki. Written informed consents were obtained from the parents of patients. The inclusion criteria of BM patients were in accordance with the World Health Organization (WHO) case definition, which was also described in other studies (36). Any child with a sudden onset of fever (>38.5°C rectal or >38.0°C axillary), altered consciousness, neck stiffness, or other symptoms showing intracranial hypertension and meningeal irritation signs and with a CSF examination showing at least one of the following changes [turbid CSF appearance, leukocytosis >100 ×10^6^ cells/L, and leukocytosis of 10–100 ×10^6^ cells/L with either an elevated protein level (>100 mg/dl) or decreased glucose level (<40 mg/dl)] was considered as a probable case. Any child that was confirmed laboratory-wise with the identification of a bacterial pathogen in the CSF or blood and with the above-mentioned clinical symptoms and signs was considered as a proven case. All the proven and probable BM patients included in this study received appropriate treatment. BM cases with congenital (e.g., inner ear malformations or dermal sinus tracts) or acquired (e.g., head injuries or basal skull fractures) anatomical defects and with serious or chronic infections of the adjacent regions (e.g., sinusitis and mastoiditis) were excluded. BM patients with primary (e.g., antibody deficiency) or secondary immunodeficiency (e.g., immunosuppressive therapy) were also excluded.

Fresh CSF was obtained via lumbar puncture and was placed in a sterile vial. Only those CSF samples in which the red blood cell (RBC) counts were zero in the routine hospital examination were chosen. Peripheral blood was drawn from a vein and was placed in a sterile anticoagulant tube with a purple cap. The collected CSF and blood samples were immediately put on ice and transported to the laboratory. Without delay, the CSF cells and supernatants were separated by direct centrifugation at 600 ×g (Eppendorf 5810R) for 5 min. The CSF supernatants were then transferred to a new centrifuge tube and stored at −20°C. The CSF cell pellets were then resuspended in 1 ml phosphate-buffered solution (PBS) supplemented with 3% fetal bovine serum (FBS, Gibco) and divided into equal amounts for the other experiments—for example, if CSF cells would be applied for two tests, then the cells were divided into two equal amounts. The blood samples were first centrifuged at 750 ×g (Eppendorf 5810R) for 10 min at 4°C, and the upper plasma layer of each sample was transferred to a new centrifuge tube and stored at −20°C. PBLs were collected by treating the remaining tube contents with ammonium chloride potassium lysis buffer (155 mM NH4Cl, 10 mM KHCO3, and 0.1 mM Na2EDTA; pH 7.2) to remove RBCs and resuspended in 1 ml PBS with 10% FBS for other experiments.

### Library preparation and sequencing for scRNA-seq

A mimic assay based on QUARTZ-SEQ2 (37) was adopted here for library preparations for scRNA-seq. An independent scRNA-seq library was constructed for 768 cells of each CSF or PBL sample. The primer set, eMDRT0001–eMDRT0768, was used to barcode each cell that was used in the QUARTZ-SEQ2 analysis. Specifically, processed sample cells were stained with 2 μm calcein-AM (MedChemExpress) for 30 min on ice and were resuspended in either PBS with 0.5% FBS for the next step if processed immediately or RNAlater solution (Invitrogen) for future analysis. The stained cells were passed through a 40-μm filter (Aladdin) and sorted using a MoFol XDP cell sorter (Beckman Coulter). Single cells with positive green fluorescence were sorted into two pre-prepared 384-well plates with cell lysis buffer, in which each well contained a unique cell barcoding primer. The following steps, including reverse transcription and whole-transcript amplification, were carried out in accordance with the protocols for QUARTZ-SEQ2.

For the preparation of each scRNA-seq sequencing library, 100 ng of amplified second-strand cDNA was incubated for 5 min at 32°C and fragmented into 400-bp sequences using a fragmentation module (NG305, TIANGEN). After purification, the fragmented cDNA was end-repaired (NG302, TIANGEN) and ligated (NG303, TIANGEN) with 0.35-μm truncated sequence adaptors (rYshapeP5-rYshapeP7LT series in QUARTZ-SEQ2). The library was further amplified and purified under the conditions described in the QUARTZ-SEQ2 protocols.

The library DNA was then sequenced using a HiSeq X Ten sequencing system (Illumina). The sequencing strategy used for the Chromium Single Cell 3′ v3 library (10x Genomics) was recreated here because it had a similar library structure to the QUARTZ-SEQ2 library. Sequencing primers X126, X899, and X908 (the sequences are shown in **Supplementary Table S2**) were used for Read1, Read2, and Index1, respectively. The sequence specifications were as follows: Read1, 151 cycles; Index1, eight cycles; and Read2, 151 cycles. The pooled raw data from one lane were demultiplexed into FASTQ files of each sample according to the index sequence in the Cell Ranger software package (10x Genomics). Clean data were generated after reads containing polluted adapters or Ns and low-quality reads were removed using the fastp package. The extraction of cell barcodes and unique molecular identifiers (UMIs), that of reads mapping to human genome GRCh38.p13, and the generation of a cell count matrix were processed in the combined workflow of UMI tools, STAR, Subread, and Samtools (https://github.com/CGATOxford/UMI-tools/blob/master/doc/Single_cell_tutorial.md).

### Library preparation and sequencing for bulkRNA-seq

To process CSF cells for bulkRNA-seq, the total RNA of the prepared cells was extracted using an RNAprep Pure Cell Kit (DP430, TIANGEN) if the TCC was over 100 × 10^6^/L or an RNAprep Pure Micro Kit (DP420, TIANGEN) if the TCC was below 100 × 10^6^/L. For processing PBLs, the total RNA of 100 ul of processed cells was extracted by RNAprep Pure Cell Kit for bulkRNA-seq.

Fifty nanograms of total RNA were then reverse-transcribed into complementary DNA (cDNA) using the primer X910 (the sequences are shown in **Supplementary Table S2**). The steps were performed using the same reactions and conditions described in the scRNA-seq method, except that the extraction of cell barcodes was not needed here.

### Data analysis of scRNA-seq and bulkRNA-seq

A secondary analysis of scRNA-seq and bulkRNA-seq was mainly carried out in the R environment. scRNA-seq data were analyzed using the Seurat package, including data normalization, feature selection of genes with high variability, linear dimension reduction, t-distributed stochastic neighbor embedding (t-SNE) clustering, and feature gene identification. Harmony was used in integrative clustering for multiple scRNA-seq data. The general cell types were annotated in the SingleR package through comparison with the Human Primary Cell Atlas Data database from the Celldex package and were further specified and confirmed with related knowledge from published papers (38–43) according to the identified feature genes of cell clusters. Differentially expressed genes (DEGs) in the transcriptomic comparison were analyzed using the DESeq2 package. Gene ontology (GO) enrichments were analyzed in the clusterProfiler package. Cell trajectory and pseudotime analyses were carried out using the Monocle 2 package. Cell–cell communication analysis was performed using CellPhoneDB in a Python environment. Ligand–receptor interactions expressed in more than 10% of the cells in the corresponding subclusters were considered relevant. Interactions with *P*-values <0.05 between two cell types were considered true connections.

### Reverse transcription and quantitative real-time polymerase chain reaction

Total RNA extraction from CSF cells and PBLs used the same method as described in the bulkRNA-seq library preparation. First-strand cDNA was generated from 50 ng of total RNA using FastKing RT Kit (KR116, TIANGEN). Quantitative real-time polymerase chain reaction (RT-qPCR) was performed using SYBR Green on a Roche LightCyler 480 system. The final 20-μl reaction mixture contained 300 nm of each primer, 1 μl of cDNA, and 10 μl of GoTaq qPCR Master Mix (Promega). All reactions were run in triplicate. RT-qPCR was initiated with a 3-min hot-start denaturation step at 95°C and followed by 40 cycles at 95°C for 15 s and then at 60°C for 1 min. *ACTB* (encoding β-actin) was employed as a reference gene. The relative expression level of the gene of interest was determined by comparing its cycle threshold (CT) value to the reference gene using the formula 2^-ΔCT^. The primer sequences of *TREM2*, *SLCO2B1*, and *ACTB* are listed in **Supplementary Table S3**.

### Flow cytometry and cell immunostaining

All operations in this section were performed at low temperature. To evaluate PMN and MN populations, processed CSF cells or PBLs were stained with 1 μg/ml Hoechst 33342 for 5 min, then fixed in PBS containing 1% paraformaldehyde, and checked through an Influx flow cytometer (BD). For cell immunostaining, processed CSF cells or PBLs were blocked for 15 min in PBS containing 0.5% bovine serum albumin (Sigma-Aldrich, A1933) and 3% FBS and then incubated for 30 min with anti-VSIG4 (4 μg/ml, Santa Cruz, sc-53977), anti-CD1C (2 μg/ml, NOVUS, NBP2-62220), or equal concentrations of isotype-matched control antibodies. After washing three times with PBS containing 3% FBS, the suspension was incubated with 2 μg/ml Alexafluor 488-conjugated goat anti-mouse antibody (4408S, Cell Signaling) for 30 min in PBS containing 0.5% bovine serum albumin and 10% goat serum (B900780, Proteintech) and then incubated for another 5 min with 1 μg/ml Hoechst 33342. The cells were washed three times and fixed in PBS containing 1% paraformaldehyde. Cellular fluorescence was measured using an Influx flow cytometer, and data were analyzed using FlowJo software.

### Enzyme-linked immunosorbent assay of cytokines

Frozen CSF supernatants and blood sera were thawed at 4°C. Interleukin-8 (CXCL8), pigment epithelium-derived factor (PEDF), and osteopontin (OPN) levels of CSF supernatants and OPN levels of blood sera were measured by commercially available sandwich-type Enzyme-linked immunosorbent assay (ELISA) (R&D Systems) following the manufacturers’ instructions.

### Statistical analysis

Statistical analysis was performed using GraphPad Prism. Student’s *t*-test was used to evaluate significant differences between two datasets. Chi-square tests were used to evaluate the contingency of cases. A significant effect was indicated by a *P*-value < 0.05.

## Results

### Study cohort

**Figure 1:**
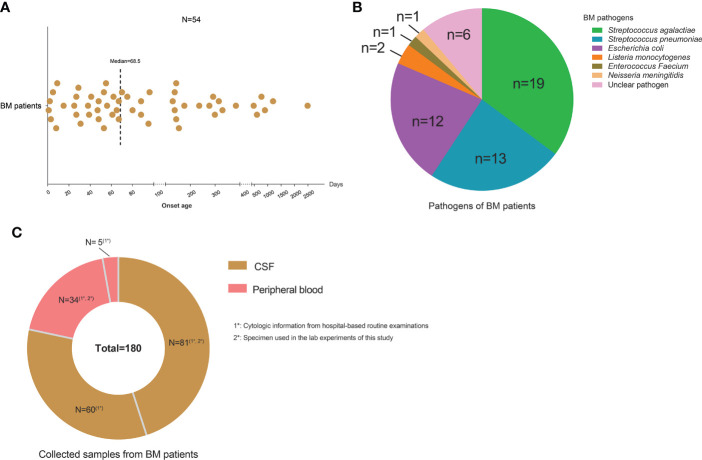
Characteristics of the study cohort. **(A)** Scatter plot showing the onset ages of bacterial meningitis (BM) patients collected in the study. One point represents one patient. **(B)** Pie chart showing the pathogens of BM patients and cases for each type of pathogen. **(C)** Pie chart showing the sample numbers of cerebrospinal fluid and peripheral blood collected from BM patients.

Fifty-four BM patients were chosen for the study from the Department of Infectious Diseases of Beijing Children’s Hospital. Their age of onset ranged from 1 to 2,490 days, with a median of 68.5 days (**Figure 1A**). Nineteen patients were infected by *Streptococcus agalactiae*, 13 patients by *Streptococcus pneumonia*, 12 patients by *Escherichia coli*, two patients by *Listeria monocytogenes*, and two patients were infected by *Enterococcus faecium* and *Neisseria meningitidis*, respectively. The remaining six patients were diagnosed by typical symptoms and CSF examinations without identified pathogens (**Figure 1B**). A total of 141 CSF and 39 peripheral blood samples were collected from the BM patients along with their cytologic information from routine hospital examination reports (**Figure 1C**). Among them, 81 CSF samples and 34 blood samples were used for multiple laboratory tests in this study. All samples were processed separately and had no cross-contamination. Detailed information about BM patients, CSF and peripheral blood samples, and tests performed on the samples is shown in **Supplementary Table S1**.

### Classifying CSF samples and their represented BM onset conditions

Because of the occurrence of CSF pleocytosis in BM, TCC is a key parameter in the diagnosis of BM. The normal TCC value of CSF in children is in the range of 0–15 × 10^6^/L. The intervention of clinical treatment for BM patients results in a continuous decline in the TCC value of CSF. Based on the above-mentioned knowledge, we classified the 141 CSF samples and their represented BM onset conditions by analyzing their hospital-based cytologic data from routine examination reports, including TCC values, PMN and MN cell proportions as well as their corresponding BM disease durations.

**Figure 2:**
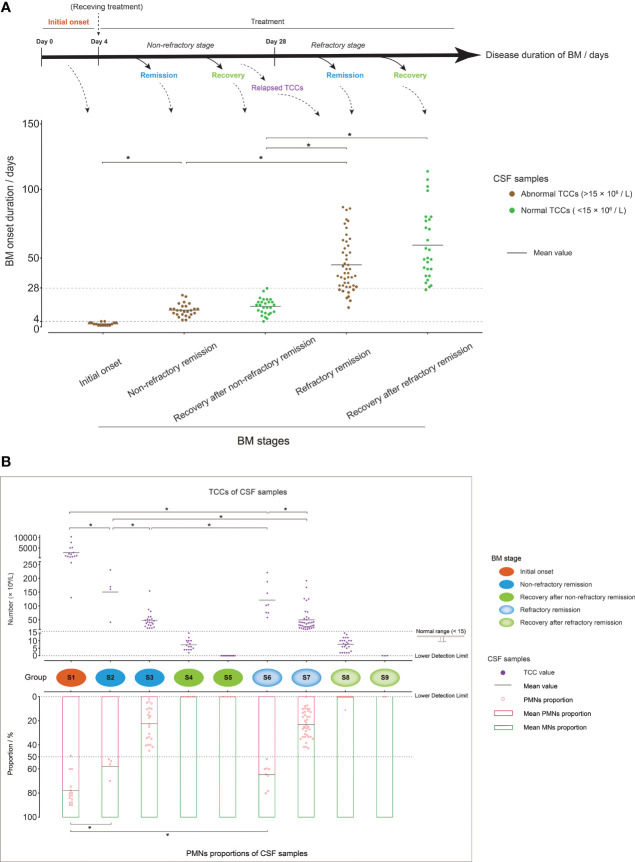
Grouping of cerebrospinal fluid (CSF) samples. **(A)** Mixed dot histogram showing the five groups of CSF samples: initial onset, non-refractory remission, recovery after non-refractory remission, refractory remission, and recovery after refractory remission. Each point represents the disease duration of bacterial meningitis (BM) corresponding to each CSF sample. The range of disease duration corresponding to the point in each group is shown in the schematic diagram above the image. The points with abnormal total cell counts (TCCs) (>15 × 10^6^/L) are marked in brown, and those with normal TCCs (<15 × 10^6^/L) are marked in green. **(B)** Composite dot histograms showing the nine groups (S1–S9) of CSF samples. The total cell count values and polymorphonuclear proportions of each CSF sample are represented by the purple dots and the pink hollow dots that are shown in the upper and lower plots, respectively. Group S1 represented the initial onset stage of BM, and groups S2/S3, S4/S5, S6/S7, and S8/S9 represented the non-refractory remission, recovery after non-refractory remission, refractory remission, and recovery after refractory remission stages of BM, respectively. *t-test, *p* < 0.05.

According to the BM disease durations and whether TCC values were in a normal range or not, the 141 CSF samples were found to represent three basic BM stages: initial onset (disease duration was within the first 4 days after BM occurrence with abnormal CSF TCC values, and the patient had not received clinical intervention or had only very recently received treatment), remission (disease duration was greater than 4 days with abnormal CSF TCC values, and the patient was under full clinical treatment intervention), and recovery (normal CSF TCC values after clinical treatment). As an acute infectious disease, the typical disease duration of BM usually do not exceed 1 month under clinical treatment (44). Therefore, the CSF samples in the remission stage were further divided into non-refractory and refractory remission, in which the corresponding disease durations were less than or greater than 28 days, respectively, during which time BM patients experienced satisfactory or unsatisfactory therapeutic effects, respectively. A recent study (45) showed that BM patients with unsatisfactory therapeutic effects presented with abnormally increased CSF TCC; in our study, some CSF of BM patients with disease durations less than 28 days showed clearly relapsed TCC values, such as C25, C40, and C97, and were classified into the “refractory remission” group. Similarly, CSF samples from BM patients in the recovery stage were divided into “recovery after non-refractory remission” or “recovery after refractory remission” groups, in which the latter group also included patients with a disease duration less than 28 days but who had recovered from a refractory condition (**Figure 2A**).

Through a further analysis of the TCC values and PMN and MN proportions in the CSF samples in each stage, the 141 CSF samples were further assigned to nine groups, which were represented by S1–S9 (**Figure 2B**). S1 represented the BM initial onset stage, in which the CSF had a higher TCC (average: 2,764 × 10^6^/L) and a higher proportion of PMN cells (average: 78%) than the other groups. The CSF samples from BM patients in the non-refractory remission stage were divided into groups S2 and S3, in which samples with a PMN cell proportion over 50% were classified as S2, and samples with a PMN cell proportion below 50% were classified as S3. The CSF samples in group S2 also had higher TCC values than those in group S3. The CSF samples in S4 and S5 represented the recovery stage after non-refractory remission. In S4, TCCs were in the normal range, and PMN cells had almost disappeared. In S5, TCCs further declined and were marked as zero in the reports of routine clinical examination as their value was lower than the detection limit. The CSF samples from patients in the BM refractory remission stage were divided into groups S6 and S7, which had similar cell characteristics to S2 and S3, respectively. S8 and S9 represented samples from patients in the recovery stage after refractory remission, which had cell characteristics similar to S4 and S5, respectively.

PMN and MN cells (MN cells are also referred to as PBMCs) are two of the main cell populations of PBLs. CSF pleocytosis is primarily caused by PBL recruitment across the blood–brain barrier, which implies that CSF cells and PBLs have a similar population composition (30). To verify the PMN and MN cell information of the CSF samples provided by the routine clinical examination reports, we checked the cell population compositions of CSF samples in the different groups through flow cytometry (FCM) (**Supplementary Figure S1**). The analysis revealed that CSF cell populations had similar compositions as PBLs, and the proportion of PMN and MN cells in different groups were in accordance with the data from routine examination reports.

### Cell heterogeneity of CSF in BM patients by scRNA-seq

**Figure 3:**
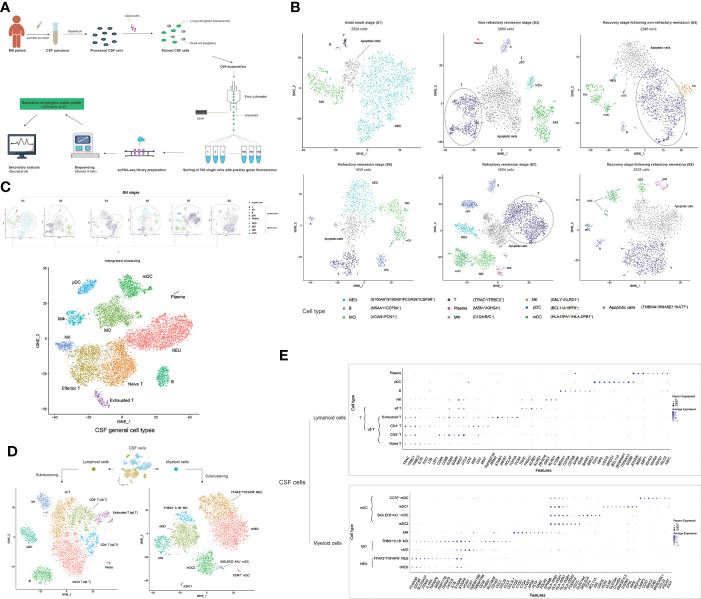
scRNA-seq reveals the cell heterogeneity of the cerebrospinal fluid (CSF) in bacterial meningitis (BM) progression. **(A)** Schematic diagram showing the scRNA-seq workflow used in this study. **(B)** t-SNE plots show the cell heterogeneity of CSF in the initial onset (S1), non-refractory remission (S3), recovery after non-refractory remission (S4), refractory remission (S6 and S7), and recovery after refractory remission (S8) stages of BM. **(C)** Combined t-SNE plots show the general 11 cell types of CSF, which are achieved from the integrated clustering of normal cells (apoptotic cells are removed) in BM S1, S3, S4, S6, S7, and S8 stages. **(D)** t-SNE plots show the subclustered nine lymphoid cell types (left) and nine myeloid cell types (right) of CSF. **(E)** Combined bubble plots showing the feature genes of nine lymphoid cell types (upper) and nine myeloid cell types (lower) of CSF identified by scRNA-seq. Shades of blue represent the relative abundance (the higher the abundance, the bluer the color), and bubble sizes represent the expression level (the higher the expression, the larger the size) of each gene. In these t-SNE plots, one point represents one cell, and different cell clusters are marked by different colors. One cell cluster represents one cell type, and the name is marked on the side.

A total of 33 CSF samples were analyzed by scRNA-seq, including four samples in S1 (C100, C103, C121, and C131), seven samples in S3 (C65, C96, C101, C106, C107, C122, and C126), four samples in S4 (C77, C102, C119, and C139), four samples in S6 (C47, C56, C58, and C59), nine samples in S7 (C55, C57, C60, C61, C66, C67, C71, C72, and C114), and five samples in group S8 (C62, C69, C73, C80, and C129), which covered the main disease conditions and key time points in BM development. CSF sample C121 was repeatedly constructed with four scRNA-seq libraries. CSF samples C114 and C131 were each repeatedly constructed with two scRNA-seq libraries. Each of the remaining CSF samples was constructed with one scRNA-seq library. The process for constructing an independent scRNA-seq library as well as sequencing and analysis is shown in **Figure 3A**.

The scRNA-seq data of CSF samples from patients in the same BM onset stage were merged and analyzed. Cells with more than 50 identified genes and less than 40% mitochondrial genes were selected for the subsequent analysis (**Supplementary Figure S2**). We obtained single-cell transcriptomes of 2,524 cells in S1, 2,896 cells in S3, 2,246 cells in S4, 1,930 cells in S6, 3,854 cells in S7, and 2,537 cells in S8 stages. We first performed t-SNE clustering and feature-gene identification of CSF cells in each stage by using the R package Seurat and cluster annotations by using SingleR (**Figure 3B** and **Supplementary Figure S3**). Nine immune cell types, including NEU (S100A8^+^, S100A9^+^, and FCGR3B^+^), MO (VCAN^+^ and FCN1^+^), MФ (C1QA^+^, C1QB^+^, and C1QC^+^), mDC (HLA-DPA1^+^ and HLA-DPB1^+^), T cell (TRAC^+^ and TRBC2^+^), NK cell (GNLY^+^ and KLRD1^+^), pDC (BCL11A^+^ and IRF8^+^), B cell (MS4A1^+^ and CD79A^+^), and plasma cell (MZB1^+^ and IGHG4^+^), were identified as well as apoptotic cells that expressed apoptosis-related genes (46–48) such as *TMBIM4*, *RNASE1*, and *KAT7*. Then, we removed the apoptotic cell data and performed an integrated clustering of normal immune cells (**Figure 3C** and **Supplementary Figure S4**). T cells were further clustered into three subtypes: naïve T (LTB^+^ and LEF1^+^), effector T (CCL5^+^ and GZMA^+^), and exhausted T cells (RRM2^+^ and STMN1^+^). In total, we identified 11 general cell types in the CSF during BM progression.

To specify the types of cells in the CSF in BM, we subclustered the myeloid cells (NEUs, MOs, MФs, and mDCs) and lymphoid cells (T cells, B cells, pDCs, and plasma cells), respectively. Nine myeloid cell subtypes and nine lymphoid cell subtypes (**Figure 3D**) and their specific feature genes (**Figure 3E**) were identified. At this level, we officially characterized 18 immune cell types in the CSF in BM. The 18 CSF immune cell types included two NEU subtypes—conventional NEUs (cNEUs) and FFAR2^+^TNFAIP6^+^ NEUs—both of which commonly expressed the genes *FCGR3B*, *CSF3R*, *SLC25A37*, and *S100A8/A9*, while FFAR2^+^TNFAIP6^+^ NEUs also highly expressed *FFAR2*, *IL1RN*, *TNFAIP6*, and *G0S2*. Two MO subtypes, conventional MOs (cMOs), and THBS1^+^IL1B^+^ MOs, commonly featured *S100A8/A9*, *VCAN*, *FCN1*, and *CD36* gene expression, while THBS1^+^IL1B^+^ MOs featured *THBS1*, *SLC39A8*, *CCL2/4/20*, and *CCL3L1* expression. One MФ cell type expressed the genes *C1QA/B/C*, *LYVE1*, *APOE*, *PLTP*, *DAB2*, *FOLR2*, and *GPNMB*. Four mDC subtypes (49)—mDC2s, SIGLEC6^+^AXL^+^ mDCs, type I mDCs (mDC1s), and CCR7^+^ mDCs—commonly had a high expression of *HLA-DPA1/B1* and *HLA-DQA1/A2/B1*. mDC2s specifically had a high expression of *FCER1A*, *CD1C*, and *CLEC10A*; SIGLEC6^+^AXL^+^ mDCs specifically had a high expression of *SIGLEC6*, *TCF4*, *BCL11A*, *AXL*, and *UGCG*; mDC1s highly expressed *XCR1*, *CLNK*, *CLEC9A*, *C1orf54*, *DNASE1L3*, *CADM1*, *CPVL*, *IRF8*, and *WDFY4*; and CCR7^+^ mDCs highly expressed *LAMP3*, *CCR7*, *BIRC3*, *CD83*, *MARCKSL1*, *CCL22*, *FSCN1*, and *IDO1*. The four αβ T cell subtypes—naïve, CD8^+^, CD4^+^, and exhausted T cells—commonly had a high expression of *TRAC*, *TRBC1*, and *TRBC2*. Naïve T cells expressed the genes *IL7R*, *TCF7*, *LTB*, and *LEF1*; CD8^+^ T cells expressed *CD8A/B* and *GZMH*; CD4^+^ T cells expressed *CD4*, *MAF*, *MIAT*, and *TNFRSF1B*; and exhausted T cells expressed *RRM2*, *STMN1*, *MKI67*, *PCLAF*, *TOP2A*, and *PCNA*. One γδ T cell type exhibited *TRGC1*, *KLRG1*, and *ZBTB16* gene expression; one NK cell type exhibited *GNLY*, *KLRD1*, and *CTSW* expression; one B cell type exhibited *MS4A1*, *CD79A/B*, *IGHD*, *BANK1*, and *BIRC3* expression; one pDC type expressed *TCF4*, *IRF8*, *UGCG*, *MPEG1*, *BCL11A*, *SERPINF1*, *TSPAN13*, and *GZMB*; and one plasma cell type exhibited *MZB1*, *XBP1*, *IGHG1/3/4*, *IGLC2*, and *IGKC* expression. Among these cell types, THBS1^+^IL1B^+^ MOs and FFAR2^+^TNFAIP6^+^ NEUs shared the expression of *FAM177B*, *TNFAIP3*, *CXCL8*, and *IL1B*. MФs and cMOs shared *TMEM176A* and *TMEM176B* expression. MФs, cMOs, and THBS1^+^IL1B^+^ MOs shared *CD163* expression. *GZMA*, *NKG7*, and *CCL5* were universally expressed on CD8^+^ T, exhausted T, γδ T, and NK cells. γδ T cells and NK cells shared the expression of *TRDC* and *KLRB1*. Plasma and B cells shared the expression of *IGHM*. Plasma cells and pDCs shared *JCHAIN* expression. *CD4* was also highly expressed on pDCs.

### The population compositions of CSF cells and their changes in BM progression analyzed by scRNA-seq

After characterizing the cell types of CSF in BM, we further analyzed the population compositions of the CSF cells in different BM stages to reveal the trends of the CSF cell population during BM progression. The 18 immune cell types, which were identified by scRNA-seq, were demultiplexed into their original BM stages (**Supplementary Figure S5**). In general, the scRNA-seq-based results showed that decreased CSF TCCs were accompanied by a decreased proportion of PMN cells (NEUs) and an increased proportion of MN cells (T cells, MOs, MФs, mDCs, pDCs, B cells, and plasma cells) in BM progression, which was in accordance with the hospital-based data shown in **Figure 2B**. Furthermore, the results also revealed that decreased CSF TCCs were accompanied by a decreased proportion of myeloid cells (NEUs, MOs, MФs, and mDCs) and an increased proportion of lymphoid cells (T cells, B cells, pDCs, and plasma cells) in BM progression (**Supplementary Figure S6**).

**Figure 4:**
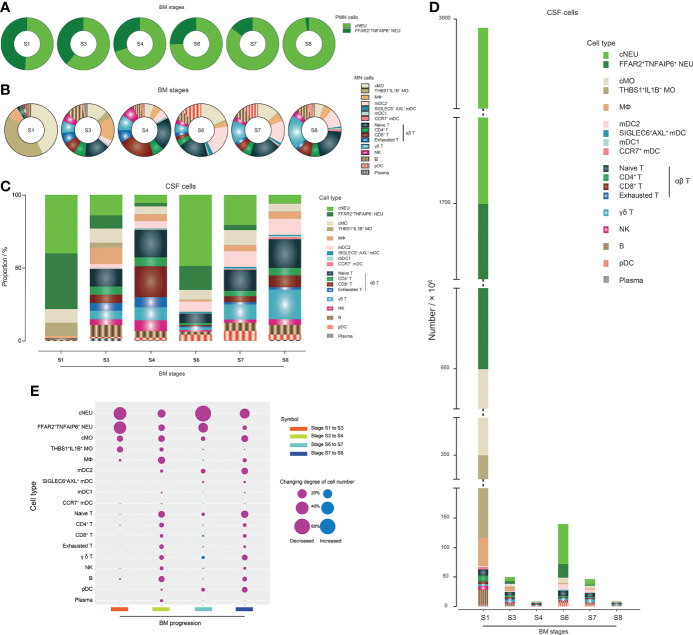
scRNA-seq reveals the population compositions of cerebrospinal fluid (CSF) cells in bacterial meningitis (BM) progression. **(A, B)** Pie charts showing the population compositions of (PMNs) **(A)** and mononuclear (MN) cells **(B)** in the BM S1, S3, S4, S6, S7, and S8 stages. Slices in the pie represent different cell types and are marked with different colors. The size of the slice represents the proportion of the corresponding cell type in the whole. **(C)** Bar chart showing the population compositions of CSF cells in BM S1, S3, S4, S6, S7, and S8 stages, which include all PMN and MN cell types. Pieces in the bar represent different cell types and are marked with different colors. The height of the piece represents the proportion of the corresponding cell type in the whole. **(D)** Bar chart showing the quantities of different CSF cell types in the BM S1, S3, S4, S6, S7, and S8 stages. Pieces in the bar represent different cell types and are marked with different colors. The height of the piece represents the quantity of the corresponding cell type. **(E)** Bubble plot showing the quantitative changing degrees of different CSF cell types during the processes of BM progression from stage S1 to S3, stage S3 to S4, stage S6 to S7, and stage S7 to S8. The higher the changing degree, the larger the bubble size. Red and blue bubbles represent decreased and increased changing numbers, respectively.

We first analyzed the cell population profile of PMN (**Figure 4A**) and MN (**Figure 4B**) cells of CSF in different stages of BM. cNEUs and FFAR2^+^TNFAIP6^+^ NEUs were found in the CSF at equal proportions in the initial stage of BM, and the proportion of FFAR2^+^TNFAIP6^+^ NEUs continued to decrease in the remission stage to the recovery stage. In the case of groups with similar TCCs, PMN cells had fewer FFAR2^+^TNFAIP6^+^ NEUs with extended disease duration, such as between groups S3 and S7 or S4 and S8. In the MN cell type, cMOs and THBS1^+^IL1B^+^ MOs were the primary cells (approximately 40% for each), and lymphoid cells had the lowest proportion (approximately 8%) in the initial onset stage of BM. The CSF samples from S3, when BM progressed, showed that the proportion of T and B cells increased to approximately 50 and 10%, respectively, while MOs decreased to approximately 16%. The MN cells in S6 and S7 samples had similar relative proportions as in S3, except that S6/S7 MN cells had a higher proportion of mDC2s (approximately 15%) and pDCs (approximately 10%), respectively, compared to those of S3 MN cells (3 and 1%, respectively). To verify this phenomenon, we extracted the scRNA-seq data of each sample in BM stages S3, S6, and S7 and found that the proportions of scRNA-seq-identified mDC2s and pDCs in S6 and S7 samples were indeed significantly higher than those of S3 (**Supplementary Figure S7**). T cells were the dominant MN cell types in S4 and S8 and accounted for approximately 75 and 60%, respectively. Among all BM stages, the CSF had relatively stable proportions of MФs and B cells, and plasma cells were relatively more abundant in the CSF of S3 compared with other stages.

The population profile of cells in the CSF (**Figure 4C**) and the quantitative characteristics of each cell type (**Figure 4D**) in BM in different stages were then analyzed, which revealed how the population profile of CSF cells changed during the progression of BM. With further calculations (**Figure 4E**), we could see that, in the period from initial onset to remission, the number of cNEUs, FFAR2^+^TNFAIP6^+^ NEUs, cMOs, and THBS1^+^IL1B^+^ MOs clearly declined. For BM patients with satisfactory therapeutic effects, the process from remission to recovery was accompanied by decreased numbers of all cell types with a relatively constant proportion. In contrast, in the process from remission to recovery of BM patients with unsatisfactory therapeutic effects, cNEU, cMO, mDC2, naïve T cell, γδ T cell, and pDC proportions declined more sharply than other cell types. We also analyzed the CSF TCC decline in the refractory remission stage. The number of cNEUs clearly decreased, and FFAR2^+^TNFAIP6^+^ NEUs, cMOs, and mDC2s also decreased considerably, while the number of CD8^+^ T and γδ T cells increased in this process, which negatively contributed to the CSF TCC decline.

### FFAR2^+^TNFAIP6^+^ NEUs and THBS1^+^IL1B^+^ MOs presented positive correlations with the intensity of inflammatory responses in CSF during BM

Among the published studies using scRNA-seq to study the characteristics of CSF cells in CNS diseases, one is on HIV (29), four are on MS (24–27), and one is on BrM (28), with one MO cluster and one NEU cluster reported. In the CSF cells of patients with MS, MOs were also CSF-derived and expressed *CD14* (26). In our study, we reported two MO clusters (cMOs and THBS1^+^IL1B^+^ MOs) and two NEU clusters (cNEUs and FFAR2^+^TNFAIP6^+^ NEUs). These four cells commonly expressed conventional *S100A8* and *S100A9* genes. cMOs and THBS1^+^IL1B^+^ MOs expressed conventional *VCAN*, *FCN1*, and *CD14* genes. cNEUs and FFAR2^+^TNFAIP6^+^ NEUs expressed conventional *FCGR3B* and *CSF3R* genes. THBS1^+^IL1B^+^ MOs and FFAR2^+^TNFAIP6^+^ NEUs also expressed multiple inflammatory cytokines, such as *TNFAIP3*, *TNFAIP6*, *CXCL8*, *IL1B*, *TNF*, and *CCL20* (**Supplementary Figure S8**). This indicated that cMOs, THBS1^+^IL1B^+^ MOs, cNEUs, and FFAR2^+^TNFAIP6^+^ NEUs in this study were in the scope of conventional MOs and NEUs, but THBS1^+^IL1B^+^ MOs and FFAR2^+^TNFAIP6^+^ NEUs were more likely two cell types with higher expressions of inflammatory cytokines that have not been reported before. We further analyzed the scRNA-seq data of CSF cells in other studies and found that the MO and NEU cells (S100A8^+^ and S100A9^+^) in the CSF of HIV and MS patients expressed low levels of THBS1^+^IL1B^+^ MO and FFAR2^+^TNFAIP6^+^ NEU-related feature genes (**Supplementary Figures S9, S10**). This meant that THBS1^+^IL1B^+^ MOs and FFAR2^+^TNFAIP6^+^ NEUs were two novel cell types discovered in this study, which participated in CNS immunity in BM. We then performed further analyses to characterize these two cell types.

**Figure 5:**
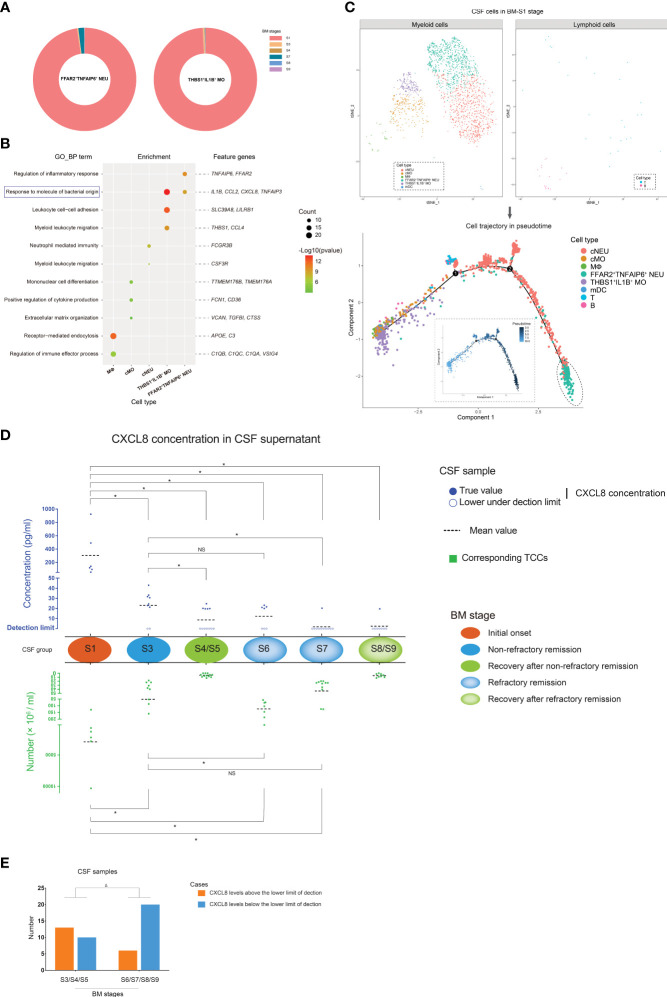
The characteristics of FFAR2^+^TNFAIP6^+^ NEUs and THBS1^+^IL1B^+^ monocytes (MOs) in bacterial meningitis (BM) progression. **(A)** Pie charts showing the quantitative distribution of FFAR2^+^TNFAIP6^+^ NEUs (left) and THBS1^+^IL1B^+^ MOs (right) in BM S1, S3, S4, S6, S7, and S8 stages, which are identified by scRNA-seq. Slices in the pie represent different BM stages and are marked with different colors. The size of the slice represents the quantitative proportion in the whole. **(B)** Bubble plot showing the Gene Ontology [GO; “biological process” (BP) aspect] enrichments targeting the feature genes of MФs, cMOs, cNEUs, FFAR2^+^TNFAIP6^+^ NEUs, and THBS1^+^IL1B^+^ MOs. Green to red indicate the log *P*-value of each GO term from low to high (the more significant the *P*-value, the redder the color) (scaled). Bubble sizes indicate the number of genes enriched for each GO-BP term. **(C)** Combined t-SNE plots (upper) and cell trajectory plots (lower) show the pseudotime analysis of the cell types in the BM S1 stage. In the cell trajectory plots, the inner and outer plots demonstrate the cell trajectory results by pseudotime and cell types, respectively. The FFAR2^+^TNFAIP6^+^ NEUs, which are located in the root state, are emphasized with dashed circles. **(D)** Composite dot histograms show the CXCL8 levels in the supernatant (upper panel) of CSF samples and corresponding total cell counts (TCCs) (lower panel) in different BM stages. CXCL8 levels above or below the lower detection limit are marked by solid blue spots or blue circles, respectively. TCC values are represented by green square points. **(E)** Bar chart showing the number of CSF samples in which the CXCL8 levels are above or below the lower detection limit in the BM non-refractory remission/recovery and refractory remission/recovery stages. *t-test, *p* < 0.05. Δ, chi-square test; *p* < 0.05. NS, no significance.

Through analyzing the distribution of scRNA-seq-identified FFAR2^+^TNFAIP6^+^ NEUs and THBS1^+^IL1B^+^ MOs in each BM stage, we found that they were abundant in the CSF of the BM initial onset stage (**Figure 5A**). In addition, compared with stage S3, fewer FFAR2^+^TNFAIP6^+^ NEUs and THBS1^+^IL1B^+^ MOs (**Supplementary Figure S11**) were identified by scRNA-seq at stage S6 and S7, even though the CSF of stage S6 had higher numbers of PMN cells and TCCs. This indicated that FFAR2^+^TNFAIP6^+^ NEUs and THBS1^+^IL1B^+^ MOs were preferentially found in the CSF with short disease durations and might undertake the main activities of an anti-bacterial infection response since pathogens would be removed under antibiotic treatment with the extension of disease duration.

We conducted a GO enrichment analysis on biological process (GO-BP) targeting the feature genes of FFAR2^+^TNFAIP6^+^ NEUs, THBS1^+^IL1B^+^ MOs, cNEUs, cMOs, and MФs, as they were the predominant cell types in the CSF from the BM initial stage (**Figure 5B**). The results showed that the feature genes of FFAR2^+^TNFAIP6^+^ NEUs and THBS1^+^IL1B^+^ MOs, such as *IL1B*, *CCL2*, *CXCL8*, and *TNFAIP3*, had a unique enrichment in the immune response to bacterial molecules compared with other cell types. In addition, the pseudotime analysis of CSF cell subtypes in the BM initial stage (**Figure 5C**) showed that FFAR2^+^TNFAIP6^+^ NEUs were located in the root state of trajectory evolution, which implied that FFAR2^+^TNFAIP6^+^ NEUs appeared earlier in the CSF than other cell types at the beginning of BM.

According to these findings, it was predicted that FFAR2^+^TNFAIP6^+^ NEUs and THBS1^+^IL1B^+^ MOs positively correlated with the intensity of the inflammatory response in CSF during BM. We measured the CXCL8 expression levels in the CSF supernatants, which was a common feature gene of FFAR2^+^TNFAIP6^+^ NEUs and THBS1^+^IL1B^+^ MOs, during the initial onset, remission, and recovery stages of BM (**Figure 5D**). The result showed that CSF in the initial onset stage contained significantly higher CXCL8 levels than the CSF in other stages. The CSF samples in the non-refractory remission stage of group S3 contained higher CXCL8 levels than in the refractory remission stage of group S7. The CXCL8 levels in the CSF did not differ between groups S3 and S6, although the CSF samples in S3 had lower TCCs. Group S7 represented abnormal BM conditions, and the CXCL8 content of group S7 CSF samples was low, similar to that of normal CSF samples. In addition, the CSF samples from groups S6, S7, and S8, with extended disease durations, contained more cases in which the CXCL8 concentrations were below the lower detection limit than those from groups S3, S4, and S5 with short disease durations (**Figure 5E**). All these findings indicated that quantitative changes in FFAR2^+^TNFAIP6^+^ NEUs and THBS1^+^IL1B^+^ MOs remained consistent with the CXCL8 level changes of CSF in BM progression.

### CSF cells of BM patients with unsatisfactory therapeutic effects featured higher proportions of mDC2s and pDCs than those of BM patients with satisfactory therapeutic effects

**Figure 6:**
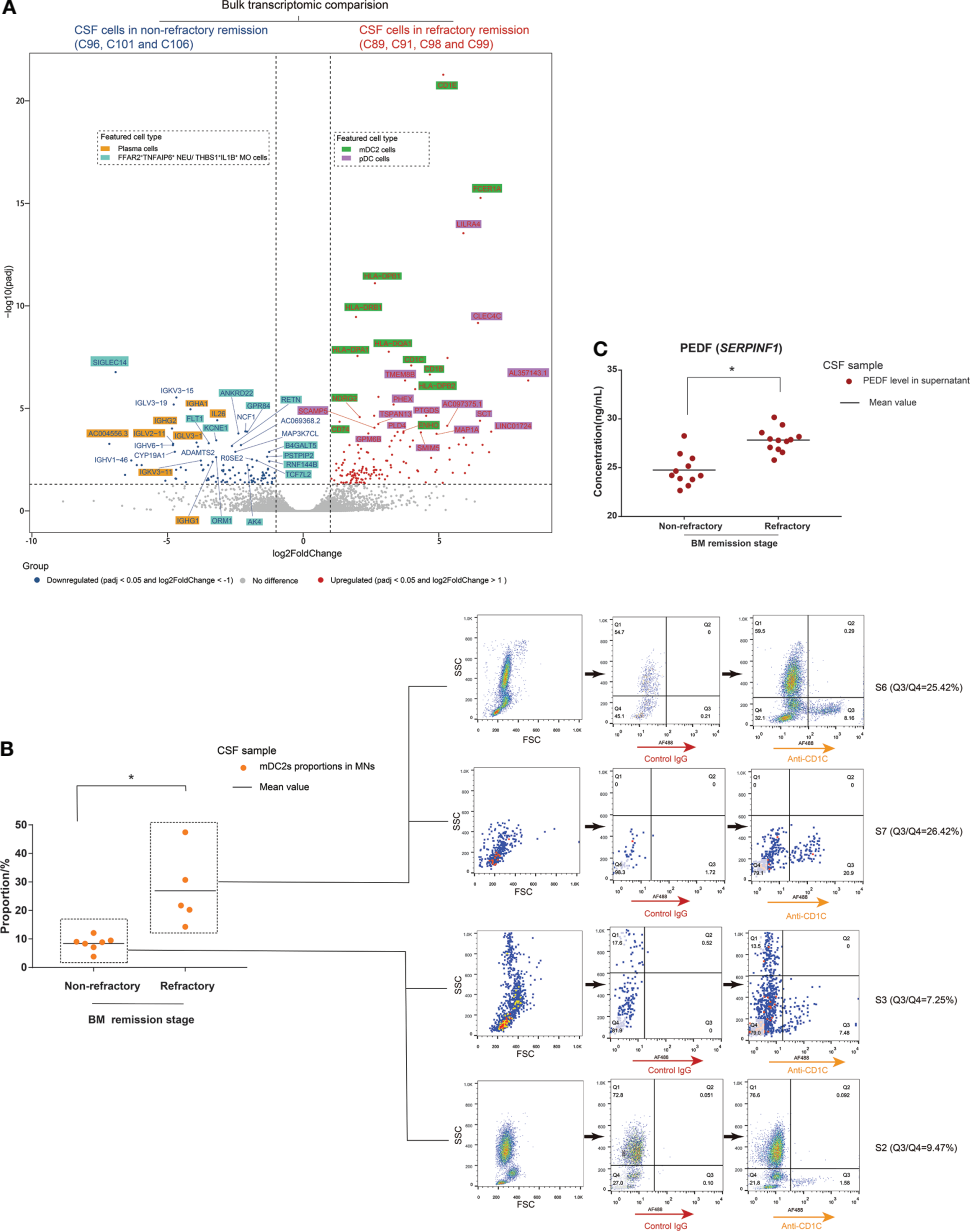
Cerebrospinal fluid (CSF) cells in the bacterial meningitis (BM) refractory remission stage feature higher proportions of mDC2s and pDCs than those in the BM non-refractory remission stage. **(A)** Volcano plot showing the DEGs identified by bulk transcriptomic comparison between CSF cells in the BM non-refractory and refractory remission stages. The x-axis shows log twofold change (log2FC) values, and the y-axis shows negative log_10_ adjusted *p*-values (padj). Each gene is represented by a single spot. Significantly downregulated (log_2_FC <-1 and padj <0.05) and upregulated (log_2_FC ≥1 and padj <0.05) DEGs are represented by blue and red spots, respectively. The thresholds of log_2_FC and padj are plotted with dashed lines. The names of downregulated and upregulated genes with top padj values are listed next to the corresponding spots. The background colors of the gene names represent the featured CSF cell types that highly express the corresponding genes. **(B)** Combined dot histogram (left) and pseudocolor images (right) show the proportions of mDC2s in mononuclear (MN) cells of CSF samples in BM non-refractory and refractory remission stages. mDC2s are represented by the cell subset with CD1C-positive (CD1C^+^) expression within MNs. In the dot histogram, each point represents the proportion of the CD1C^+^ cell subset in MNs of a CSF sample. The representative CD1C expressions of MNs in CSF samples in BM S2, S3, S6, and S7 stages are shown in the pseudocolor images. The ratio of cell number in the Q3 (CD1C^+^) and Q4 (CD1C^-^) quadrants demonstrates the proportion of mDC2s in MNs. **(C)** Dot histogram showing the PEDF (encoded by *SERPINF1*) levels in supernatants of CSF samples in BM non-refractory and refractory remission stages. Each dot corresponds to the PEDF level in the supernatant of a CSF sample. *t-test, *p* < 0.05.

The refractory condition of BM patients during clinical treatment requires more attention. The scRNA-seq characterization of CSF cells in non-refractory compared to refractory remission stages revealed different cell heterogeneity between them. We further explored this difference. In our study, we performed bulkRNA-seq tests targeting the cells of 12 CSF samples as additional support for the scRNA-seq data. The CSF samples included two samples (C95 and C100) in S1, three samples (C96, C101, and C106) in S3, one sample (C102) in S4, one sample (C104) in S5, one sample (C91) in S6, three samples (C89, C98, and C99) in S7, and one sample (C94) in group S8. We then compared the bulk transcriptomes of CSF cells from non-refractory remission patients (C96, C101, and C106) with those from refractory remission patients (C91, C89, C98, and C99) and analyzed the representative cell types of top DEGs in combination with the scRNA-seq data (**Figure 6A**, **Supplementary Figures S12, S13**). The top downregulated DEGs were mainly expressed on plasma cells (including *IGHA1*, *IGHG1/2*, *IGLV3-1*, and *IL26*) and FFAR2^+^TNFAIP6^+^ NEUs and THBS1^+^IL1B^+^ MOs (including *ANKRD22*, *GPR84*, *RETN*, and *B4GALT5*). The top upregulated DEGs were mainly expressed on mDC2s (including *CD1E*, *FCER1A*, *HLA-DPB1*, *HLA-DRB1*, and *CD1C*) and pDCs (including *LILRA4*, *CLEC4C*, *TSPAN13*, and *TMEM8B*). Therefore, the bulkRNA-based data was in accordance with the scRNA-based data. The top upregulated DEGs had much lower adjusted *P*-values (padj) than the top downregulated DEGs, which meant that the feature difference in population compositions between CSF cells in the non-refractory and refractory remission stages was due to mDC2s and pDCs. In other words, the compositions of CSF cells in the refractory stage had a higher proportion of mDC2s and pDCs than that of the non-refractory remission stages.

We then measured CD1C expression on CSF cells through FCM immunostaining and measured the PEDF (encoded by *SERPINF1*) levels in CSF supernatants through ELISA using samples from non-refractory and refractory remission stages of BM. As marker genes, CD1C expression and PEDF levels represented the number of mDC2s and pDCs in the CSF, respectively. The results showed that the MN population of CSF from the refractory remission stage contained higher proportions of CD1C^+^ cells than that of CSF from the non-refractory remission stage (**Figure 6B**). The PEDF levels in the supernatants of CSF samples from the refractory remission stage were significantly higher than in those from the non-refractory remission stage (**Figure 6C**).

### CSF cells of BM patients with unsatisfactory therapeutic effects had different intercellular communications compared with patients with satisfactory therapeutic effects

**Figure 7:**
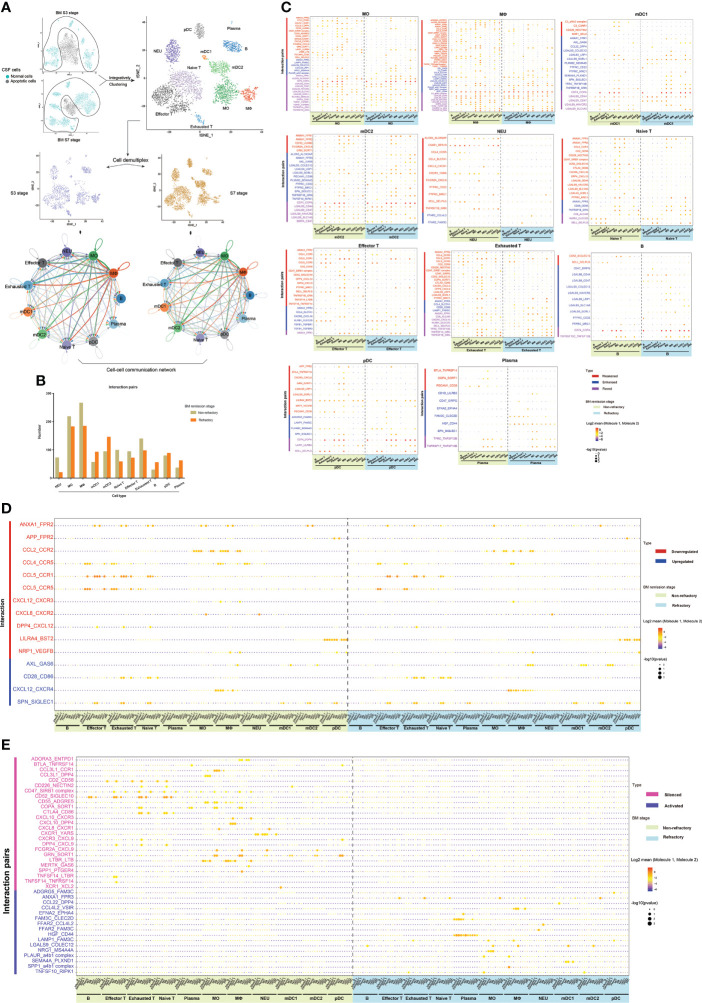
The different cell–cell communications between cerebrospinal fluid (CSF) cells in the bacterial meningitis (BM) non-refractory and refractory remission stages. **(A)** t-SNE plots showing the workflow for the analysis of intercellular communications, and network diagrams show the cell–cell interactions between each cell type of CSF in the non-refractory and refractory remission stages. In the network diagrams, the line color is the same as the cell type that expresses the ligands. The lines connect to the cell types that express the cognate receptors. The line thickness is proportional to the number of ligands when cognate receptors are present in the recipient cell type. The loops indicate autocrine circuits. **(B)** Bar chart showing the number of ligand–receptor interactions of each CSF cell type in the non-refractory and refractory remission stages. **(C)** Bubble plots showing the ligand–receptor interactions with changed levels in MOs, MФs, mDC1s, mDC2s, NEUs, naïve T cells, effector T cells, exhausted T cells, B cells, pDCs, and plasma cells between the non-refractory and refractory remission stages. The types of changed levels are marked with multiple colors: weakened (red), enhanced (blue), and roved (purple). **(D)** Bubble plot showing the downregulated and upregulated interactions in CSF cells between the non-refractory and refractory remission stages, which are marked by red and blue colors. **(E)** Bubble plot showing the silenced and activated interactions in CSF cells between the non-refractory and refractory remission stages, which are marked by pink and violet colors. In these bubble plots, bubble colors indicate the means of the average expression levels of interacting molecule 1 in cluster 1 and interacting molecule 2 in cluster 2. Bubble sizes indicate *P*-values. The stronger the interaction, the redder the color and the larger the size.

After delineating the different population compositions of CSF cells between the non-refractory and refractory remission stages, we then studied whether they had different cell–cell interactions as they were in different disease states. This analysis was performed on CSF samples from groups S3 and S7 as they had similar cell characteristics. The scRNA-seq data of CSF cells in groups S3 and S7 were reanalyzed and integratively clustered to ensure that they had consistent cell types, and intercellular communications were analyzed using the CellPhoneDB package (**Figure 7A**).

The CSF cells in groups S3 and S7 were integratively clustered into 11 general cell types: NEUs, MOs, MФs, mDC1s, mDC2s, naïve T cells, effector T cells, exhausted T cells, B cells, pDCs, and plasma cells. All cell types interacted with each other in both the non-refractory and refractory remission stages. Compared with those in the non-refractory remission stage, NEUs, MOs, MФs, naïve T cells, effector T cells, and exhausted T cells had decreased connections with other cell types, while mDC1s, mDC2s, B cells, pDCs, and plasma cells had increased interactions in the refractory remission stage of BM (**Figure 7B**).

The ligand–receptor interactions in each CSF cell type which had different levels between the non-refractory and refractory remission stages were then analyzed (**Figure 7C**). The interaction levels that differed between the two groups were represented by three categories: weakened, enhanced, and roved. Specifically, “weakened” and “enhanced” interactions meant that one cell type had this connection with fewer cell types in the refractory remission stage and with more cell types in the non-refractory remission stage. A “roved” interaction meant that one cell type had this connection with different cell types in refractory remission stages compared with those in the non-refractory remission stage. A total of 89 ligand–receptor interactions with different levels between the stages among all cell types were identified (**Supplementary Table S4**). They included cytokine receptor-related, cell–cell adhesion-related, immune response-related, immune suppression-related, and immune checkpoint-related interactions (50)—for example, LGALS9-related interactions were extensively active in the intercellular communications of MOs, MФs, mDC1s, mDC2s, and B cells but rarely in NEUs, T cells, and plasma cells.

Through a further analysis, we found 11 ligand–receptor interactions that were downregulated and four that were upregulated within CSF cells from the non-refractory remission stage compared with those from the refractory remission stage (**Figure 7D**). “Downregulated” meant that the interactions occurred between fewer cell types in the refractory remission stage than in the non-refractory remission stage, while “upregulated” meant that interactions occurred between more cell types in the refractory remission stage than in the non-refractory remission stage—for example, the ANXA1_FPR2 interaction was attenuated between connections of NEUs with effector T cells, exhausted T cells, naïve T cells, MOs, and mDC2s and between connections of MФs with MOs and NEUs in the refractory remission stage compared with the ANXA1_FPR2 interaction in the non-refractory remission stage. The AXL_GAS6 interaction was enhanced between connections of pDCs with MФs, mDC1s, and mDC2s in the refractory remission stage compared with that in the non-refractory remission stage. In addition, we also found 25 silenced and 16 activated interactions that specifically occurred in CSF cells in the non-refractory and refractory stages, respectively (**Figure 7E**)—for example, the CD52_SIGLEC10 interaction was active in the connections between T and B cells with MOs, MФs, and mDC1s in the non-refractory remission stage, but this interaction was silenced in all cells in the refractory remission stage. The HGF_CD44 interaction was silenced in CSF cells in the non-refractory remission stage but was widely activated in the communication between plasma cells and other cell types in the refractory remission stage.

### Distinct molecular hallmarks of CSF cells and PBLs against CNS infection and sepsis in sepsis-developed BM

Immunity in the CNS is considered an independent unit with special characteristics due to the presence of the blood–brain barrier and its immune privilege (51, 52). We then explored whether CSF cells and PBLs exhibited different characteristics in sepsis-developed BM.

**Figure 8:**
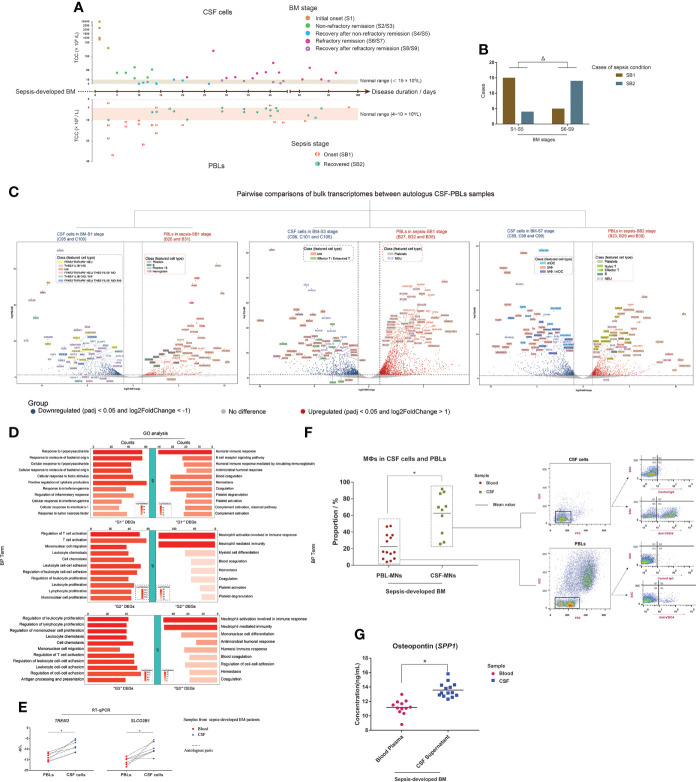
Different molecular hallmarks between cerebrospinal fluid (CSF) cells and peripheral blood leukocytes (PBLs) in sepsis-developed bacterial meningitis (BM). **(A)** Composite dot plots showing the total cell count (TCC) values and corresponding BM disease durations of 37 CSF samples (upper) and autologous PBLs (lower). Each point represents a CSF sample or PBL sample, and its TCC value is reflected on the y-axis, while the corresponding BM onset duration is reflected on the x-axis. The BM stages of each point are marked with different colors. **(B)** Bar chart demonstrating the cases of PBL samples in SB1 (brown color) and SB2 (blue–gray color) conditions, while their corresponding CSF samples are in S1–S5 and S6–S9 stages. **(C)** Volcano plots showing the differentially expressed genes (DEGs) of the pairwise bulk transcriptomic comparison between CSF cells in BM-S1 stage and PBLs in sepsis-SB1 stage (left), CSF cells in BM-S3 stage and PBLs in sepsis-SB1 stage (middle), and CSF cells in BM-S7 stage and PBLs in sepsis-SB2 stage (right). The x-axis shows log twofold change (log_2_FC) values, and the y-axis shows negative log_10_ adjusted *p*-values (padj). Each gene is represented by a single spot. Significantly downregulated (log_2_FC <-1 and padj <0.05) and upregulated (log_2_FC >1 and padj <0.05) DEGs are represented by blue and red spots, respectively. The thresholds of log_2_FC and padj are plotted with dashed lines. The names of downregulated and upregulated genes with top padj values are listed next to the corresponding spots. The background colors of the gene names represent the featured CSF cell types that highly express the corresponding genes. **(D)** Bar charts showing the terms of Gene Ontology [GO; “biological process” (BP) aspect] enrichments targeting the “G1^-^” and “G1^+^” (upper), “G2^-^” and “G2^+^” (middle), and “G3^-^” and “G3^+^” (lower) DEGs. Shades of red indicate the log *P*-value of each GO term from low to high (the more significant the *P*-value is, the redder the color) (scaled). Bar lengths indicate the number of genes enriched for each GO term. **(E)** Dot histograms showing the relative mRNA expression of *TREM2* (left) and *SLCO2B1* (right) in autologous CSF cells and PBLs by RT-qPCR. Each red spot or blue diamond point represents the gene expression in a PBL or CSF cell sample, respectively. **(F)** Combined dot histogram (left) and pseudocolor images (right) show the proportions of MФs in mononuclear (MN) cells of CSF and PBL samples. MФs are represented by the cell subset with VSIG4-positive (VSIG4^+^) expression within MNs. In the dot histogram, each red spot and yellow-green square point represents the proportion of the VSIG4^+^ cell subset in MNs of a PBL and CSF sample, respectively. Representative VSIG4 expressions in MNs of CSF and PBL samples are shown in pseudocolor images. The ratio of cell number in the Q3 (VSIG4^+^) and Q4 (VSIG4^-^) quadrants demonstrates the proportion of MФs in MNs. **(G)** Dot histogram showing the osteopontin (OPN) (encoded by *SPP1*) levels in CSF supernatants and blood plasmas. Each red spot or each blue square point represents the OPN level in the supernatant of a CSF sample or plasma of a blood sample, respectively. *t-test; Δ, chi-square test; *p* < 0.05.

Peripheral blood specimens were obtained from BM patients in this study. The sampling time interval was always 2 days or less, which meant that the autologous CSF cells and PBL samples were essentially collected at the same pathological state of BM. We collected hospital-based data from routine examination reports of blood samples to obtain the TCC values of the PBLs and then jointly analyzed the TCC changes of the autologous CSF–blood pairs during BM progression (**Figure 8A**). We found that, in the early stages of BM, the TCCs of most PBL samples were abnormal (normal range: 4–10 ×10^9^/L), which indicated an onset condition (named SB1) of sepsis. If BM patients suffered refractory conditions, the TCCs of PBLs recovered to normal in the refractory remission stage of BM. This meant that when BM patients suffered refractory BM conditions with long remission durations, their sepsis symptoms appeared resolved, which indicated a recovered condition (named SB2) of sepsis. This phenomenon was confirmed with a significant difference (**Figure 8B**).

For the 12 CSF samples analyzed for bulkRNA-seq in this study, the bulk transcriptomes of their corresponding autologous PBLs were also sequenced. According to the characteristics of sepsis-developed BM progression, we compared the bulk transcriptomic difference between pairwise CSF cells and PBLs in three groups: group I, CSF cells (C95 and C100) in the initial onset stage of BM versus PBLs (B26 and B31) in the onset stage of sepsis; group II, CSF cells (C96, C101, and C106) in the non-refractory remission stage of BM versus PBLs (B27, B32, and B35) in the onset stage of sepsis; and group III, CSF cells (C89, C98, and C99) in the refractory remission stage of BM versus PBLs (B23, B29, and B30) in the recovered stage of sepsis (**Figure 8C**). The downregulated DEGs in groups I, II, and III were marked as “G1^-^”, “G2^-^”, and “G3^-^”, while the upregulated DEGs were marked as “G1^+^”, “G2^+^”, and “G3^+^”, respectively. In addition, to characterize the representative cell types of upregulated DEGs, we constructed a scRNA-seq library of PBLs from sample B24 and acquired the single-cell transcriptomic data of 357 cells. These PBLs were identified as seven cell types (**Supplementary Figure S14**): NEUs (FCGR3B^+^, CSF3R^+^, and SLC25A37^+^), MOs (CD300E^+^, FCN1^+^, and VCAN^+^), naïve T cells (TCF7^+^, IL7R^+^, and LEF1^+^), effector T cells (CST7^+^, NKG7^+^, and GZMA^+^), B cells (VPREB3^+^, MS4A1^+^, and CD79A^+^), platelets (ACRBP^+^, NRGN^+^, PPBP^+^, and PF4^+^), and apoptotic cells (TMBIM4^+^, KAT7^+^, and RNASE1^+^).

Through analysis, we found that the top G1^-^ DEGs, such as *CXCL8*, *PI3*, *SPP1*, *CCL20*, and *CCL2*, were mainly expressed in FFAR2^+^TNFAIP6^+^ NEUs and THBS1^+^IL1B^+^ MOs and MФs of CSF (**Supplementary Figure S15**), while the top G1^+^ DEGs were mainly expressed in the platelets (including *PPBP*, *PF4*, *SPARC*, and *MYL9*) or B cells (including *CD79A*, *IGKC*, and *IGHM*) of PBLs or in both the platelets and B cells (*PDLIM1*) (**Supplementary Figure S16**). The top G2^-^ DEGs were mainly expressed in MФs (including *SPP1*, *APOE*, *C1QA*, *C1QC*, and *GPNMB*) and effector T and exhausted T cells (including *SCD*, *CCR5*, *CXCR6*, *FABP5*, and *CTLA4*) of CSF (**Supplementary Figure S17**), while the top G2^+^ DEGs were mainly expressed in NEUs (including *S100P*, *S100A8*, *IFITM2*, and *CYP4F3*) and platelets (including *MYL9*, *NGRN*, *MPIG6B*, and *TREML1*) of PBLs (**Supplementary Figure S18**). Many top G3^-^ DEGs were feature genes of MФs or mDCs (including *CLEC10A*, *CD1E*, *HLA-DQA1*, and *CLIC2*) or both (including *MRC1*, *IL18*, *ATP1B1*, and *GGTA1P*) (**Supplementary Figure S19**). Additionally, the top G3^+^ DEGs included the feature genes of platelets, naïve T cells (including *LRRN3*, *MAL*, *TCF7*, and *ABLIM1*), effector T cells (including *FGFBP2*, *ADGRG1*, and *SPON2*), B cells (*IGHD* and *PAX5*), and NEUs (including *CDA*, *S100A12*, *S100P*, and *IFIT2/3*) of PBLs (**Supplementary Figure S20**).

Additional GO-BP enrichment analyses (**Figure 8D**) showed that G1^-^ DEGs were enriched in immune responses to bacteria and G1^+^ DEGs were enriched in humoral immune response and blood coagulation. G2^-^ DEGs were enriched in T cell activation, cell chemotaxis, leukocyte proliferation, and cell–cell adhesion, while G2^+^ DEGs were enriched in NEU-related immunity, myeloid cell differentiation, and blood coagulation. G3^-^ DEGs had high enrichments in leukocyte proliferation, cell chemotaxis, cell–cell adhesion, and antigen processing and presentation, while G3^+^ DEGs were enriched in NEU-related immunity, mononuclear cell differentiation, humoral immune response, and blood coagulation.

Through transcriptomic comparisons between autologous CSF cells and PBLs, we found that CSF cells always had a high expression of MФ feature genes and PBLs had a high expression of platelet feature genes, no matter how the onset conditions and disease progression of BM and sepsis changed. When simultaneously comparing the bulk transcriptomes between CSF cells and PBLs of the 12 autologous CSF–blood pairs, the top downregulated DEGs were usually MФ feature genes of CSF, and the top upregulated DEGs were almost always platelet feature genes of PBLs (**Supplementary Figure S21**). The expression of some MФ feature DEGs in CSF cells and PBLs was then evaluated. The mRNA expression levels of *TREM2* and *SLCO2B1* in CSF cells and PBLs were measured through RT-qPCR, and it was found that CSF cells expressed significantly higher levels of *TREM2* and *SLCO2B1* than PBLs (**Figure 8E**). The level of VSIG4 in CSF cells and PBLs was measured through FCM immunostaining, and the results (**Figure 8F**) revealed that CSF MN cells had a significantly higher proportion of the VSIG4^+^ cell subgroup than PBL MN cells. To evaluate *SPP1*, we measured the concentration of OPN in multiple CSF supernatants and blood plasmas acquired from sepsis-developed BM patients, and the results (**Figure 8G**) showed that CSF supernatants contained significantly higher OPN levels than blood plasmas.

## Discussion

Infants and young children are at increased risk for BM due to their immature immune systems. The clinical course and outcomes of BM are relatively complex, which could be influenced by multiple elements such as therapeutic treatment, pathogen species, etiological factors, and individual difference. In this study, we conducted research among patients with relatively similar clinical features. To do this, we restricted the BM patients to those whose disease was caused by sepsis, with the age of the patients concentrated in infants. The pathogens were common species, in which *S. agalactiae*, *E. coli*, and *S. pneumoniae* infections accounted for most cases, which was in accordance with the BM incidence in other countries (53–55). We also grouped the CSF samples, instead of treating them as a single population, according to disease onset and progression by analyzing hospital-based data. However, our study still had room for improvement—for example, the biochemical parameters of CSF such as glucose and protein were not considered in our studies. In general, as a progressive step towards elucidating the detailed cell profile of CSF in acute CNS diseases, this study provided valuable information for understanding BM pathogenesis and significant implications for further research.

The scRNA-seq characterization of CSF cells in BM revealed a different heterogeneity and neuroinflammatory condition compared with those in other CNS diseases. In the CSF of MS and RRMS patients (25–27), T cells, especially CD4^+^ and CD8^+^ T cells, composed the majority of the cell population, and in the CSF of HIV patients (29), IL7R^+^ T and CD8^+^ T cells were the majority of the cell population. For BrM patients (28), NK cells and tumor-associated MФs were the major cells in CSF. In contrast, our study found that NEUs and MOs were the main cell types in the CSF when BM occurred. In particular, we identified that FFAR2^+^TNFAIP6^+^ NEUs and THBS1^+^IL1B^+^ MOs undertook an important role in the immune response to bacterial infection. This meant that the main neuroinflammatory response in BM patients was innate immunity, while it was cellular immunity in MS, HIV, and BrM patients. Furthermore, in the cluster comparison between CSF cells and PBLs, the BM patient samples also showed different results compared with the MS and RRMS patient samples. The CSF contained higher mDC2s in MS patients as well as mDCs, MФs, and pDCs in RRMS patients. In contrast, in BM patients, we found that FFAR2^+^TNFAIP6^+^ NEUs, THBS1^+^IL1B^+^ MOs, and MФs were higher in CSF in the initial onset of BM. Notably, in BM, MS, and RRMS patients, PBLs had higher fractions of B cells and platelets compared with the CSF, which indicated that humoral immunity and coagulation were relatively weak in the neuroinflammations of these two distinct CNS disorders. In addition, we identified exhausted T cells and the absence of Treg cells in the CSF of BM patients; however, the opposite was found in the CSF of MS and RRMS patients, which identified Treg cells with an absence of exhausted T cells. Exhausted T cells and Treg cells could suppress the regulation of effector cytotoxicity (56, 57), and the mechanism of maintaining T cell immune homeostasis in the case of hyperactivation might differ between BM and MS. In addition to exhausted T cells, SIGLEC14^+^AXL^+^ mDCs and CCR7^+^ mDCs were also identified in the CSF of BM patients but were missing in the CSF of MS and RRMS patients. As these two DC subsets have only been recently discovered, their exact functions are still unclear, and therefore their functions in BM need further study. Overall, these results indicated that the host immune system mobilized a unique cellular framework to respond to different types of neuroinflammatory disorders.

Refractory BM exhibits a relatively long disease duration similar to chronic meningitis (58). Although refractory BM has received significant attention, the causes and pathological features are still unclear. Considering the CSF cell compositions, the CSF in the refractory stage contained fewer FFAR2^+^TNFAIP6^+^ NEUs and THBS1^+^IL1B^+^ MOs, which indicated that an immune response to a bacterial infection was not the main reaction to this condition. In other words, the activities of the cells in the elevated TCCs did not focus on the clearance of pathogens. The low CXCL8 level in the CSF supernatant also indicated that the refractory stage exhibited a low neuroinflammatory intensity. Furthermore, compared with cell–cell communications in non-refractory BM, multiple ligand–receptor interactions involved in inflammatory cytokines, such as CCL4, CCL5, CXCL8, and CXCL10, were attenuated in the CSF cells in the refractory stage. All these findings indicated that the abnormal condition with an elevated TCC of CSF in the refractory stage was in a status with low-grade inflammation. Additionally, the emergence of a refractory condition was accompanied with changes of the CSF cell population compositions and, in particular, an increase of mDC2s. mDC2s enhanced the inhibitory interactions with each other and with other cell types during the refractory phase and particularly via molecular LGALS9. LGALS9 has been shown to suppress cell immunities (59, 60), and its active expression might induce mDC2s in a dysfunctional state, which could lead the host to mobilize more mDC2s to compensate for the functional deficiencies. LGALS9-related interactions are also extensively active in B cells, MOs, MФs, and mDC1s. Other inhibitory interactions were also upregulated in refractory BM—for example, CD74_COPA (59) was activated in MOs, mDC1s, mDC2s, pDCs, and B cells, TNFSF/TNFRSF pair-related interactions (61) were active in mDC2 with MOs, MФs, and mDC1s, and PTPRC_CD22 (62) was active in B cells with mDC1s and mDC2s. These indicated that the immune function of CSF cells in the refractory stage might be in an over-suppressed state, leading to a persistently abnormal disease duration. For refractory BM conditions, traditional treatment such as antibiotics may not be suitable and new therapies need to be developed.

MS, RRMS, and BrM are more commonly adult CNS diseases, and sufficient CSF can be obtained for studies. In comparison, the BM patients in our study could only provide 0.5–1.0 ml of CSF since the donors were at a very young age, and sampling too much CSF would further endanger their health. Limited cell numbers increased the experimental difficulty, but we conquered this dilemma by developing a suitable scRNA-seq method and successfully performing scRNA-seq analyses of the limited CSF cells of BM patients. In conclusion, our research not only unravels the cell heterogeneity of CSF in BM but also provides possible treatment targets for BM patients.

## Data availability statement

Raw and processed data files for scRNA-seq and bulkRNA-seq have been deposited under GEO accession number GSE163219, which includes three subseries: GSE163194, GSE163195 and GSE163196. Specifically, GSE163194 and GSE163195 were used for scRNA-seq and bulkRNA-seq of CSF cells, respectively, and GSE163196 was used for bulkRNA-seq of PBLs. The other datasets used and/or analyzed during the current study are available from the corresponding author on reasonable request.

## Ethics statement

This study was reviewed and approved by The Ethics Committee of Beijing Children’s Hospital, Capital Medical University. Written informed consent to participate in this study was provided by the participants’ legal guardian/next of kin. Written informed consent was obtained from the individual(s) and minor(s)’ legal guardian/next of kin for the publication of any potentially identifiable images or data included in this article.

## Author contributions

HHX and HJX conducted the experiments, analyzed data, made figures, and wrote the paper. YZ discussed the draft paper. LG, ZD, LL, LZ, WF, BL, BH, and TC aided for CSF and blood sampling. GL and TW designed the experiments, supervised the project, and revised the paper. All authors contributed to the article and approved the submitted version.

## Funding

This work was supported by grants from the Strategic Priority Research Program of the Chinese Academy of Sciences (XDA17010503), the National Science and Technology Major Project (2018ZX10101004003003), Beijing Natural Science Foundation (no. L202004), The Special Fund of the Pediatric Medical Coordinated Development Center of Beijing Hospitals Authority (no. XTZD20180501), Respiratory Research Project of National Clinical Research Center for Respiratory Diseases (no. HXZX-202106), and Young Talent Training Project of Beijing Hospital Authority (QML20191203).

## Acknowledgments

We are grateful to Junying Jia (Institute of Biophysics, Chinese Academy of Sciences, Beijing, China) and Deqin Feng (Institute of Microbiology, Chinese Academy of Sciences, Beijing, China) for the help with single-cell sorting by FCM.

## Conflict of interest

The authors declare that the research was conducted in the absence of any commercial or financial relationships that could be construed as a potential conflict of interest.

## Publisher’s note

All claims expressed in this article are solely those of the authors and do not necessarily represent those of their affiliated organizations, or those of the publisher, the editors and the reviewers. Any product that may be evaluated in this article, or claim that may be made by its manufacturer, is not guaranteed or endorsed by the publisher.
