# Supplementary material for: High-throughput sequencing unravels the cell heterogeneity of cerebrospinal fluid in the bacterial meningitis of children
Source: Front Immunol. 2022 Sep 2;13:872832. doi: 10.3389/fimmu.2022.872832 (PMC9478118; doi:10.3389/fimmu.2022.872832)
Supplement: Supplementary file 1 [file DataSheet_1.pdf]

Supplementary Materials

High-throughput sequencing unravels the cell heterogeneity of cerebrospinal fluid in the bacterial meningitis of children

Haihan Xiao[a, b, †], Haijuan Xiao[c, †], Yun Zhang[a], Lingyun Guo[c], Zhenzhen Dou[c], Linlin Liu[c], Liang Zhu[c], Wenya Feng[c], Bing Liu[c], Bing Hu[c], Tianming Chen[c], Gang Liu[c, *], Tingyi Wen[a, d, *]

[a]CAS Key Laboratory of Pathogenic Microbiology and Immunology, Institute of Microbiology, Chinese Academy of Sciences, Beijing 100101, China

[b]University of Chinese Academy of Sciences, Beijing 100049, China

[c]Department of Infectious Diseases, Key Laboratory of Major Diseases in Children, Ministry of Education, Beijing Children's Hospital, Capital Medical University, National Center for Children's Health, Beijing 100045, China

[d]Savaid Medical School, University of Chinese Academy of Sciences, Beijing 100049, China

*Corresponding author. E-mail: wenty@im.ac.cn

*Corresponding author. E-mail: liugangbch@sina.com

[†]These authors contributed equally to this work.

Table of contents

- **Supplementary Figures S1-S21**
- **Legends to supplementary Tables S1-S4**
- **Additional results**

Supplementary Figures

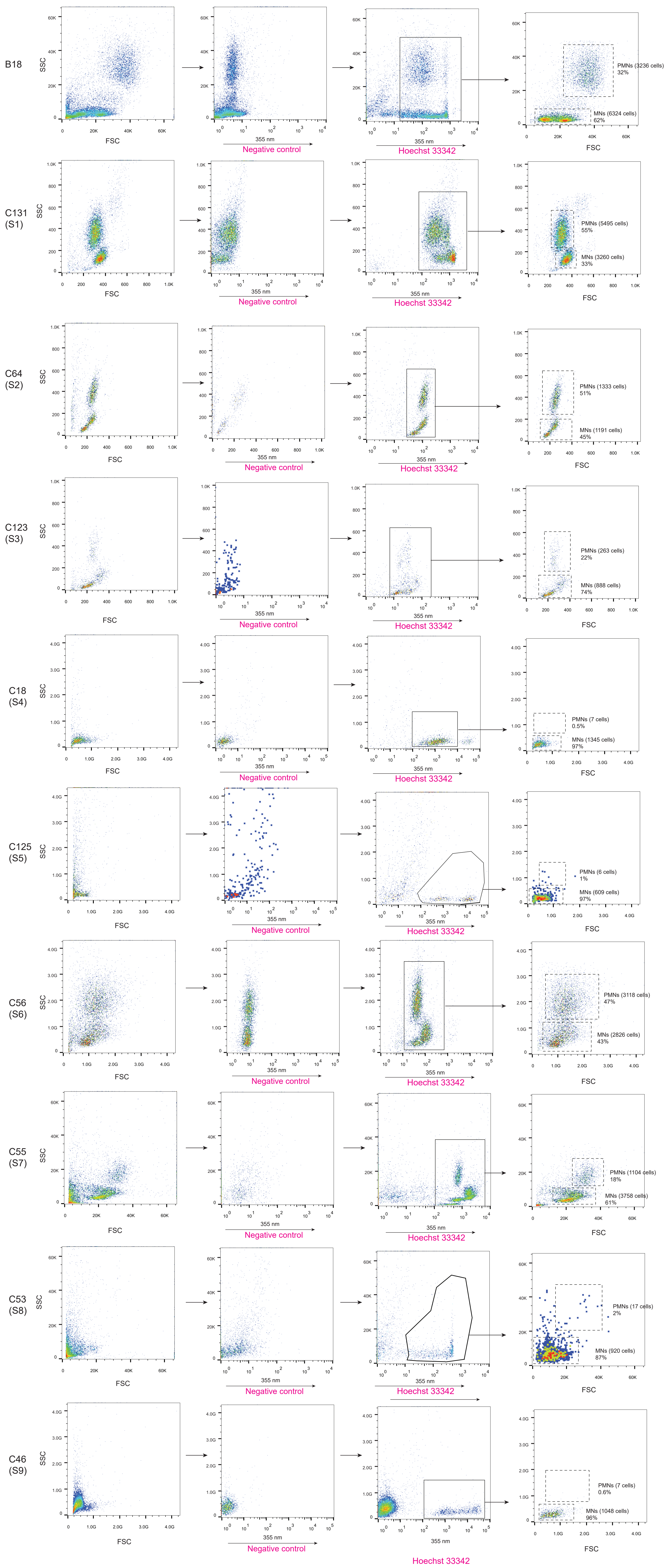

Figure S1. Pseudocolor images exhibit the PMNs and MNs populations in blood and CSF samples. The fluorescence of cells in the negative control is blank. Hoechst-33342 dye is used to stain the cell nucleus, and cells with positive fluorescence (enclosed by boxes with solid lines) are selected for displaying the PMNs and MNs populations. PMNs and MNs populations are emphasized by boxes with dashed lines. The counts and proportions of the cells in the boxes with dashed line are marked on the side.

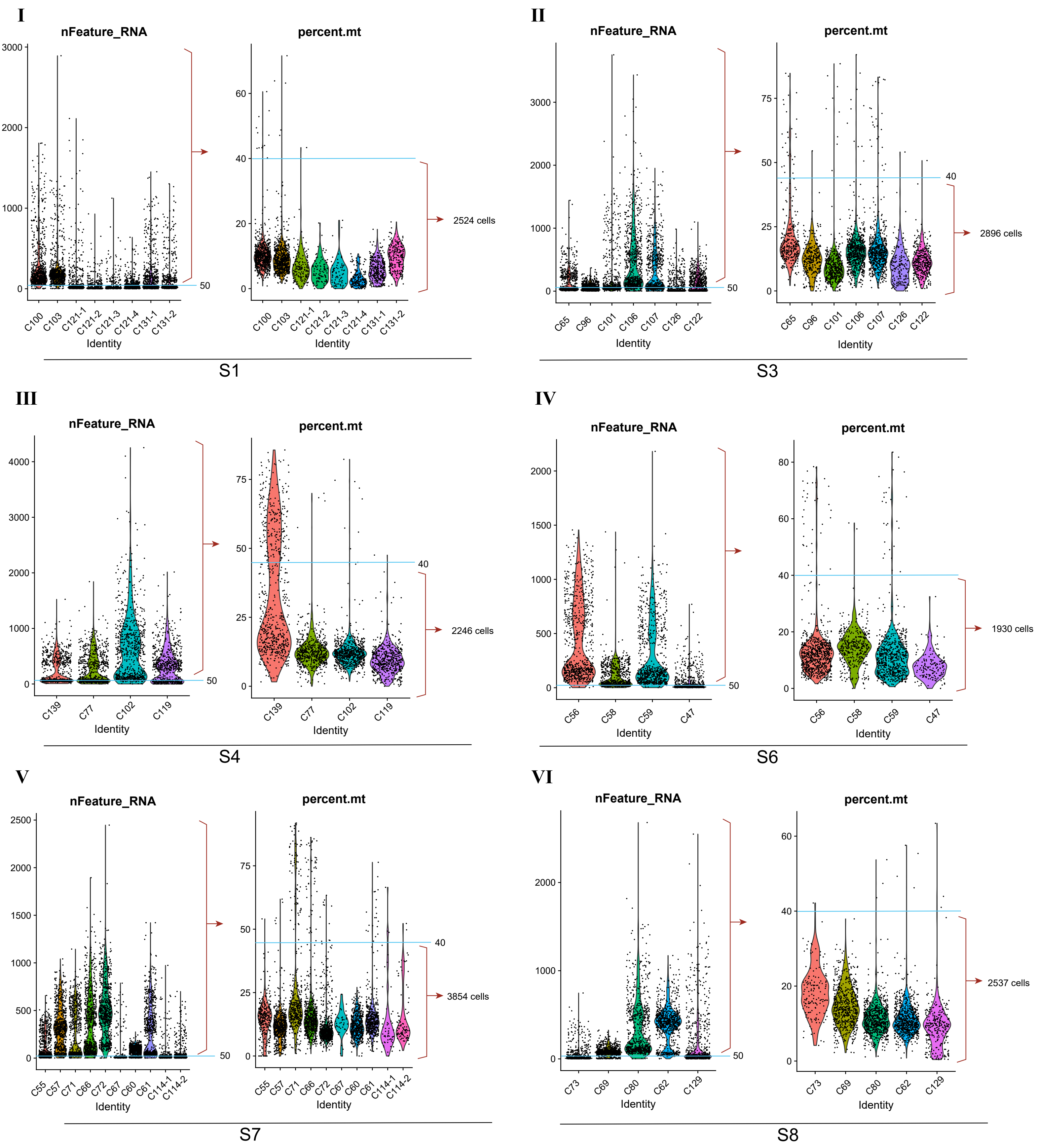

Figure S2. Violin scatterplots illustrate the number of genes (nFeature_RNA) and the percentage of mitochondrial genes (percent.mt) of each cell in scRNA-seq libraries of CSF samples in the BM S1 (I), S3 (II), S4 (III), S6 (IV), S7 (V) and S8 (VI) stages. The cell filtering criteria (more than 50 identified genes and less than 40% of mitochondrial genes), as well as the acquired cell numbers after quality control, are displayed on the images.

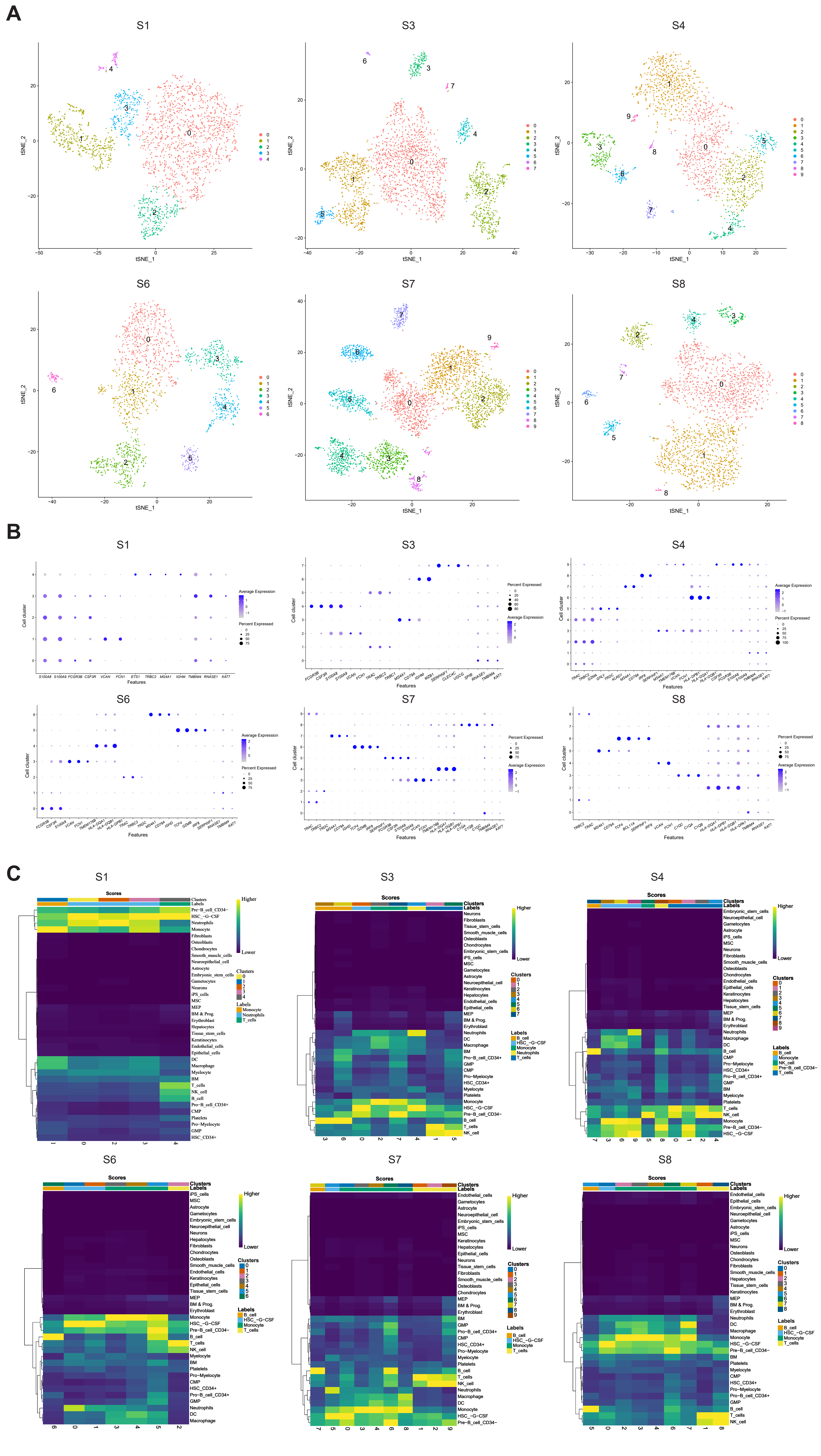

Figure S3. scRNA-seq data analysis of CSF cells in the BM S1, S3, S4, S6, S7 and S8 stages. (A) t-SNE plots show the cell clusters of CSF in each BM stage. One point represents one cell, and different cell clusters are marked by different colors. The names of clusters are marked by the numbers. **(B)** Bubble plots show the feature genes of cell clusters in each BM stage. Shades of blue represent the relative abundance (the higher the abundance, the bluer the color), and bubble sizes represent the expression level (the higher the expression, the larger the size) of each gene. **(C)** Heatmaps show the mapping results of cell clusters by alignment with human cell types in the Human Primary Cell Atlas Data database (Celldex package in R) in each BM stage. The color from dark to bright represents the matching degree from low to high.

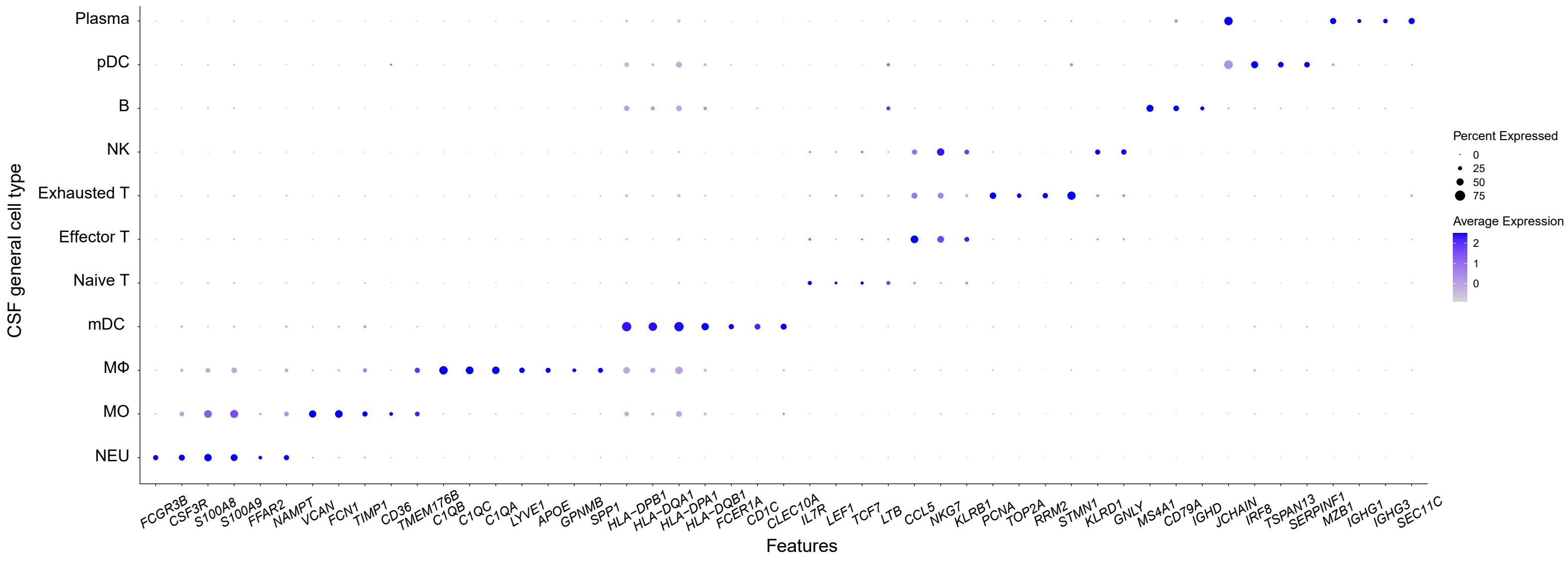

Figure S4. The bubble plot shows the feature genes of CSF general cell types identified by scRNA-seq. Shades of blue represent the relative abundance (the higher the abundance, the bluer the color), and bubble sizes represent the expression level (the higher the expression, the larger the size) of each gene.

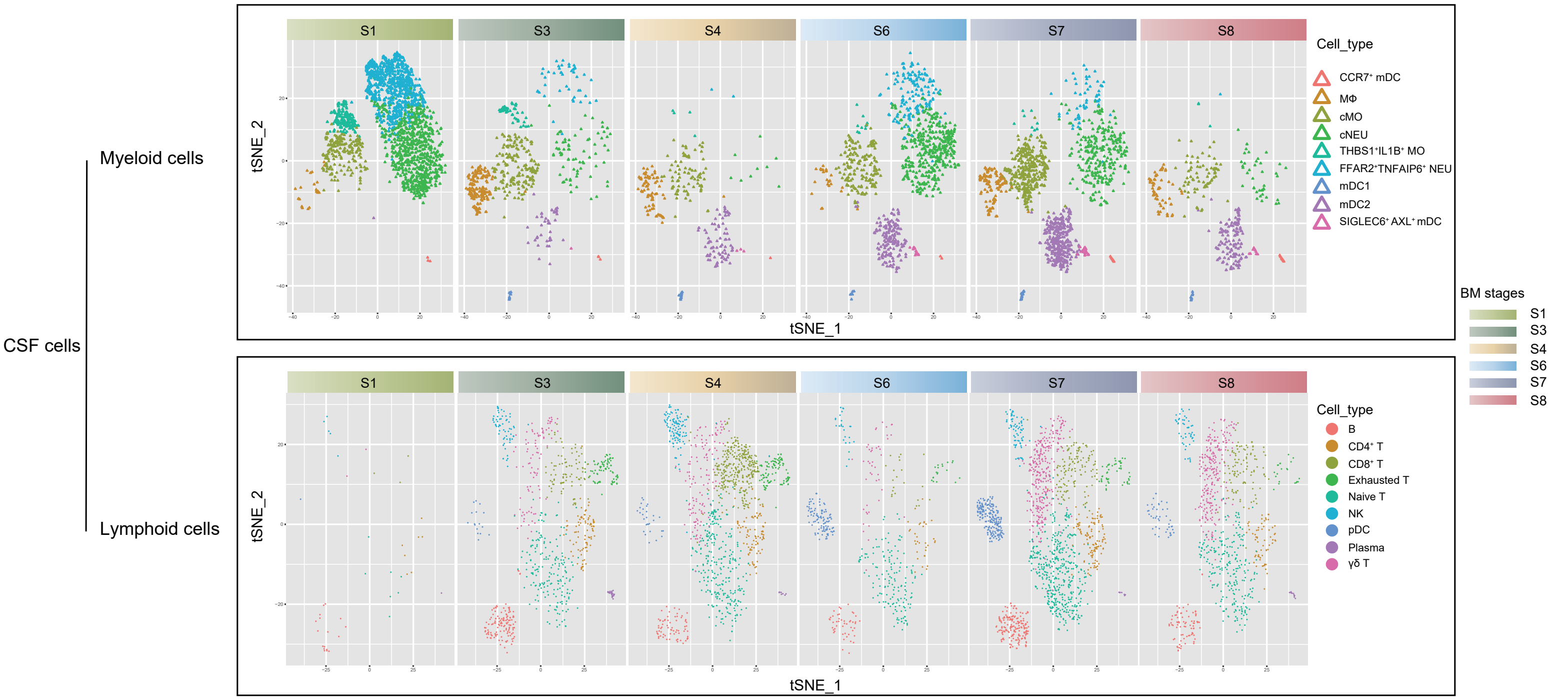

Figure S5. Combined t-SNE plots show the quantitative distribution of myeloid cell types (upper panel) and lymphoid cell types (lower panel) of CSF in different BM stages, which are identified by scRNA-seq. Each myeloid cell type is marked by multicolored hollow triangles, while each lymphoid cell type is marked by multicolored points.

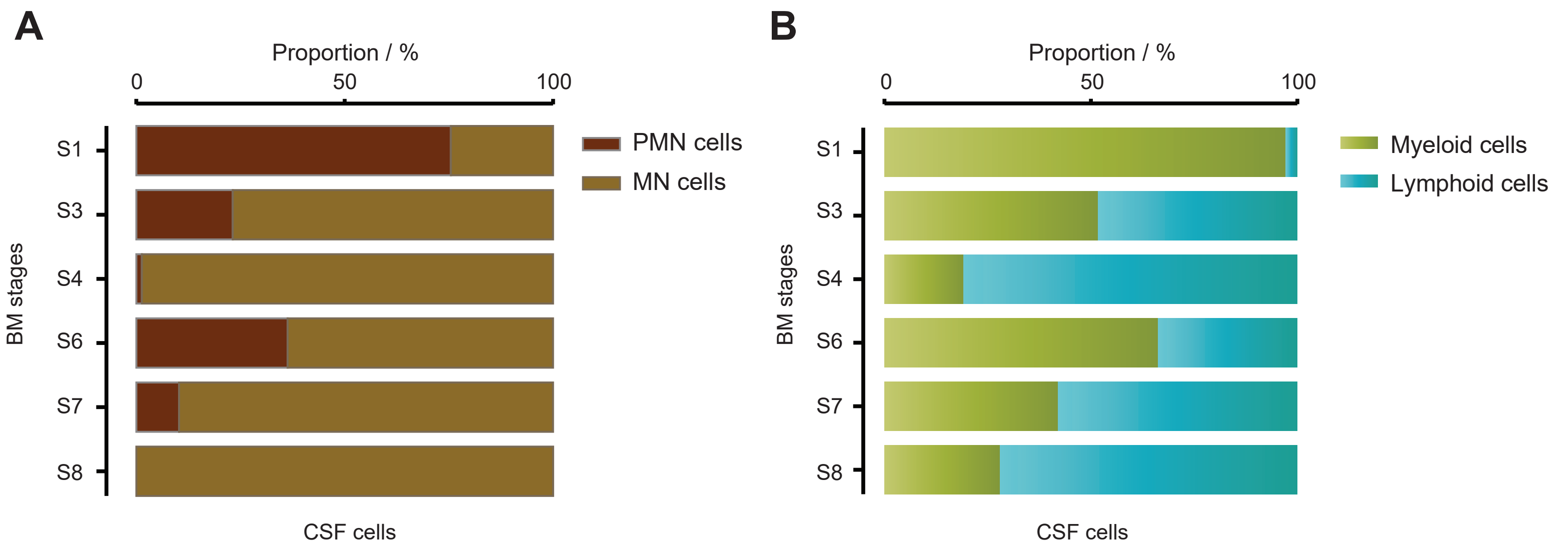

Figure S6. The basic population compositions of CSF cells in the BM S1, S3, S4, S6, S7 and S8 stages identified by scRNA-seq. (A-B) Bar chart shows the population compositions of CSF cells consisting of PMNs and MNs (A) or myeloid and lymphoid cells (B) in each BM stage. Pieces in the bar represent different cell types and are marked with different colors. The width of the piece represents the proportion of the corresponding cell type in the whole.

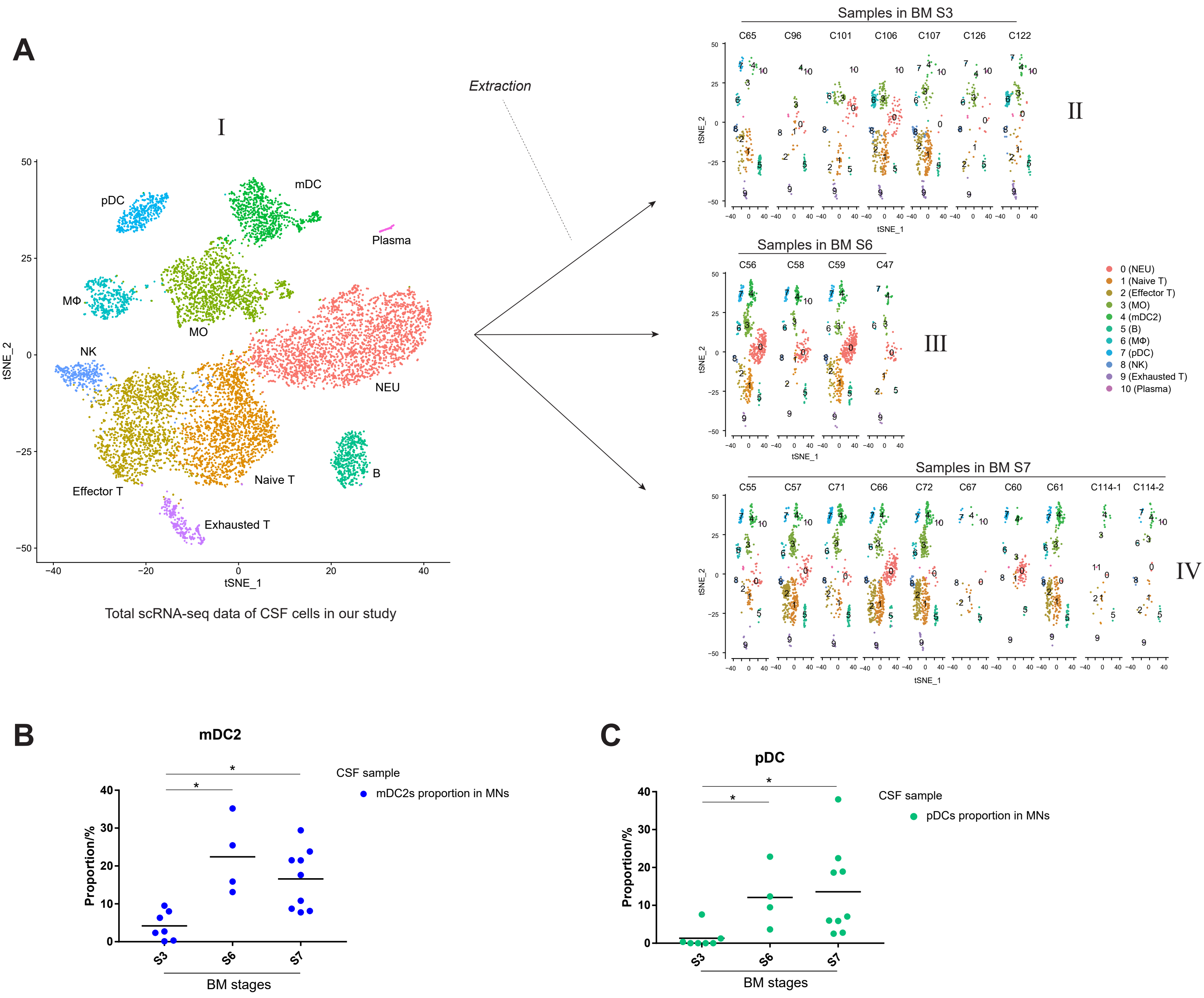

Figure S7. scRNA-seq reveals higher proportions of mDC2s and pDCs in CSF cells of BM S6 and S7 stages than in S3 stage. (A) Combined t-SNE plots demonstrate the quantitative distributions of CSF general cell types (I) in each CSF sample in the BM S3 (II), S6 (III) and S7 (IV) stages. One point represents one cell, and different cell clusters are marked by different colors. **(B-C)** Dot histograms show the proportions of mDC2s (B) and pDCs (C) in MNs of CSF samples at BM S3, S6 and S7 stages. Each blue or green spot represents the proportions of mDC2s or pDCs in MNs of a CSF sample, respectively. *: t test, $p < 0.05$.

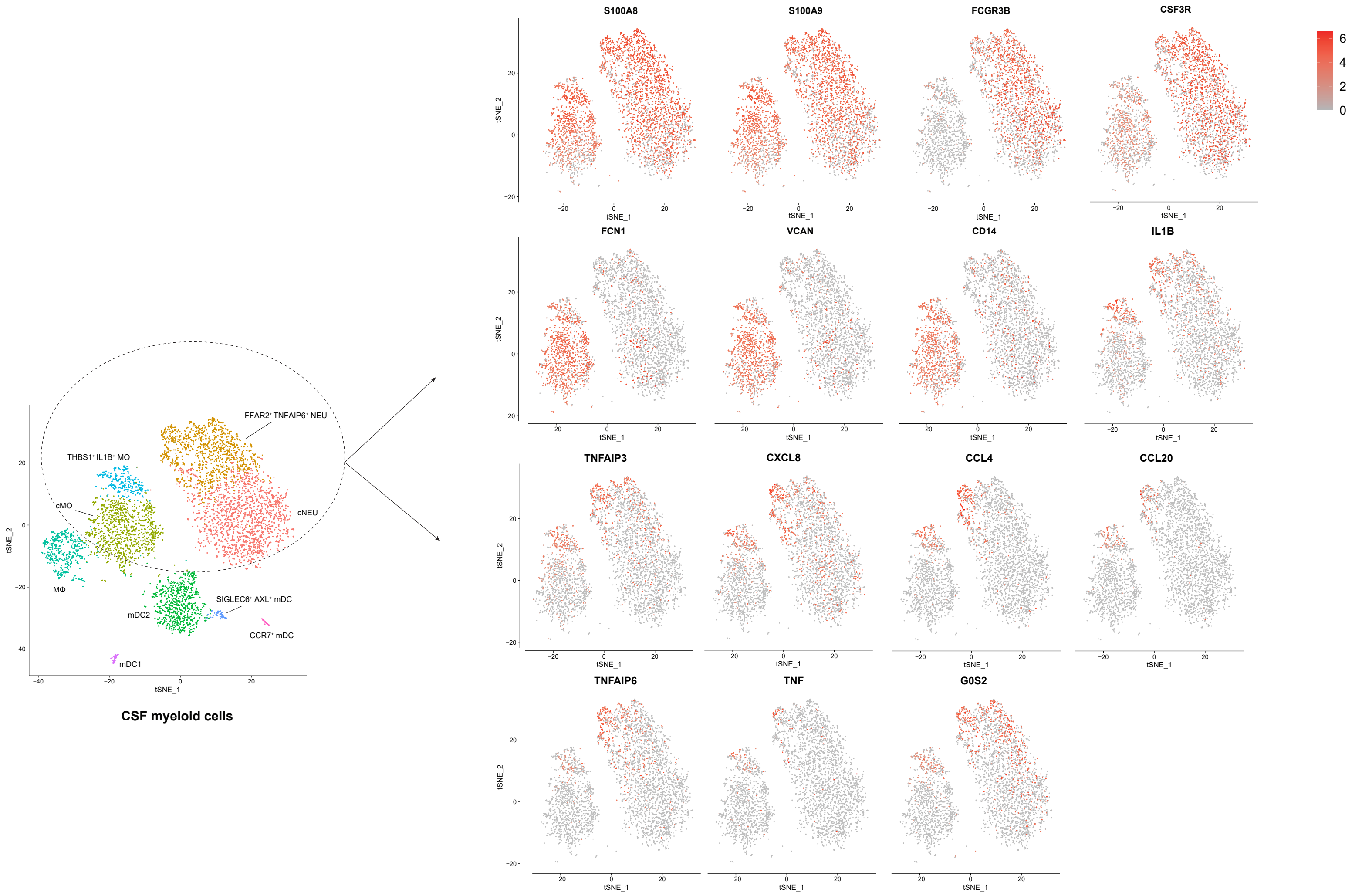

Figure S8. t-SNE plots indicate the expression of multiple genes in cNEUs, cMOs, FFAR2⁺TNFAIP6⁺ NEUs and THBS1⁺IL1B⁺ MOs. One point represents one cell. Shades of red represent the relative expression (the higher the expression, the redder the color).

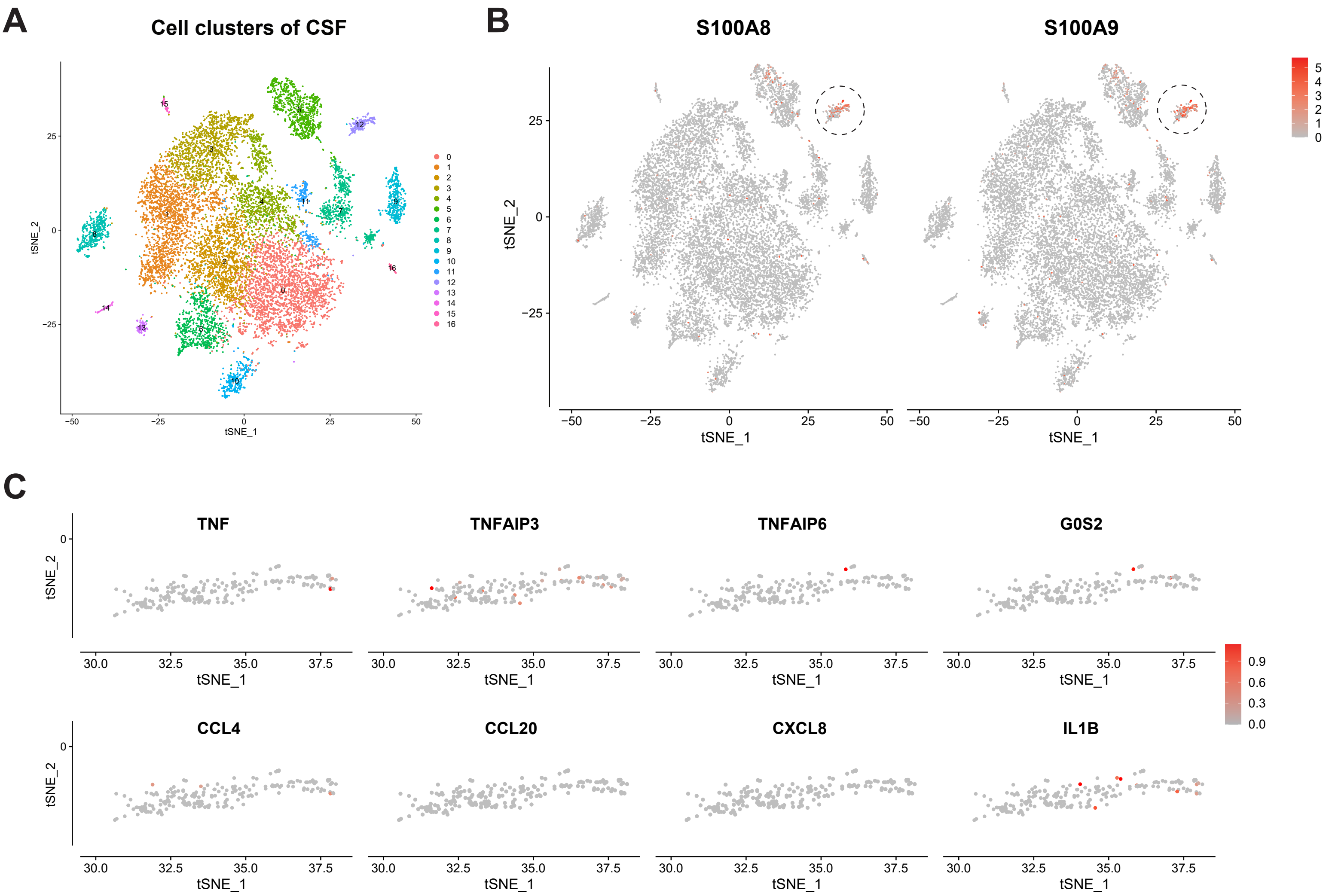

Figure S9. The expression of FFAR2^{+}TNFAIP6^{+} NEUs and THBS1^{+}IL1B^{+} MOs characteristic genes in the NEUs and MOs of CSF of AIDS patients, which is based on analysis of scRNA-seq data from a published paper (*Shelli F. Farhadian, et al. JCI Insight, 2018*). (A) t-SNE plot shows the CSF cell clusters of AIDS patients. One point represents one cell, and different cell clusters are marked by different colors. The names of clusters are marked by the numbers. **(B)** t-SNE plots indicate the expression of S100A8 (left) and S100A9 (right) in the CSF cell clusters. One point represents one cell. Shades of red represent the relative expression (the higher the expression, the redder the color). The areas with high abundance are emphasized with dashed circles. **(C)** t-SNE plots indicate the expression of TNF, TNFAIP3, TNFAIP6, G0S2, CCL4, CCL20, CXCL8 and IL1B in cluster NO. 12 of CSF cells. One point represents one cell. Shades of red represent the relative expression (the higher the expression, the redder the color).

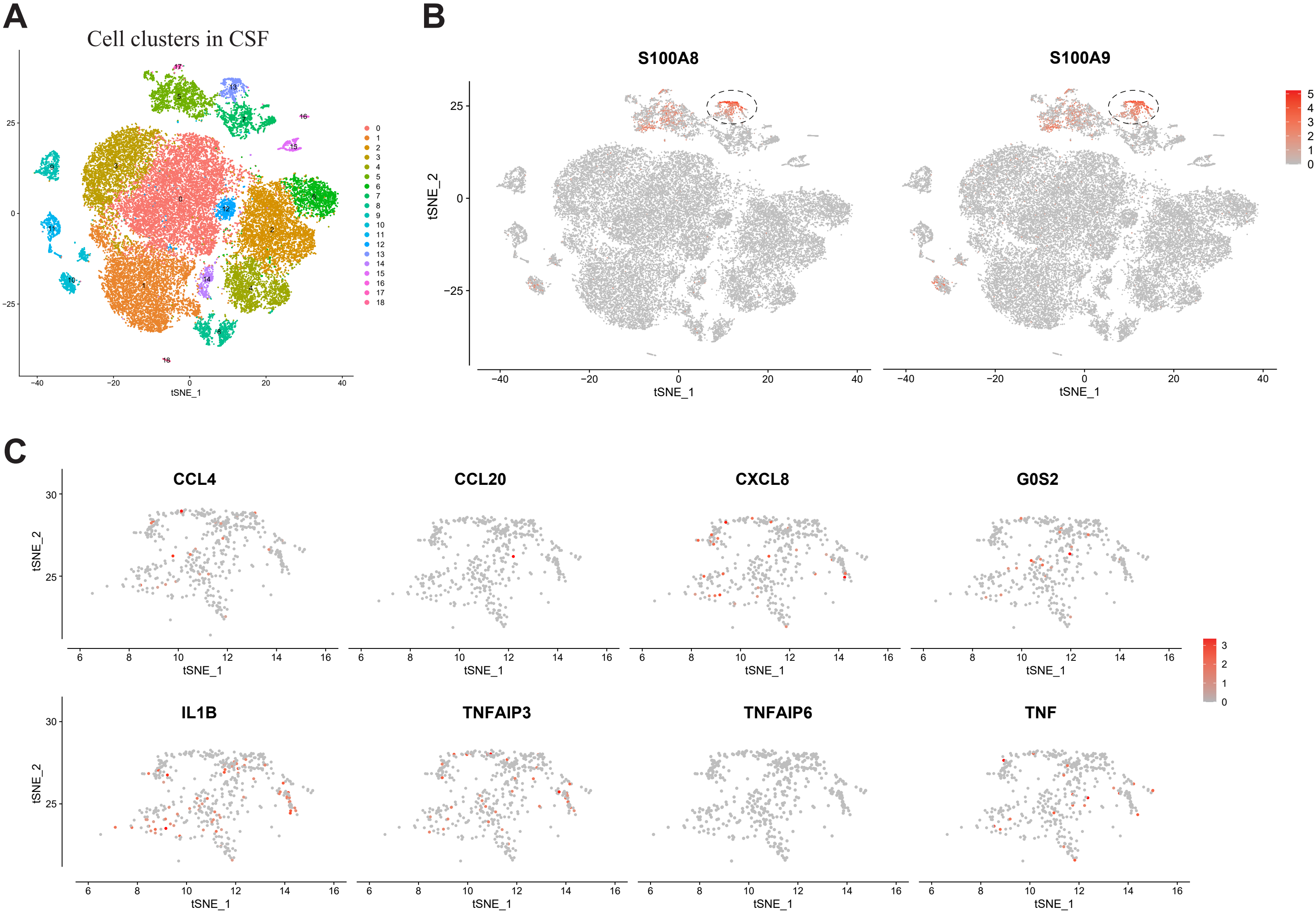

Figure S10. The expression of FFAR2^{+}TNFAIP6^{+} NEUs and THBS1^{+}IL1B^{+} MOs characteristic genes in the NEUs and MOs of CSF of MS patients, which is based on analysis of scRNA-seq data from a published paper (*David Schafflick, et al. Nature Communications, 2020*). (A) t-SNE plot shows the CSF cell clusters of MS patients. One point represents one cell, and different cell clusters are marked by different colors. The names of clusters are marked by the numbers. **(B)** t-SNE plots indicate the expression of S100A8 (left) and S100A9 (right) in the CSF cell clusters. One point represents one cell. Shades of red represent the relative expression (the higher the expression, the redder the color). The areas with high abundance are emphasized with dashed circles. **(C)** t-SNE plots indicate the expression of TNF, TNFAIP3, TNFAIP6, G0S2, CCL4, CCL20, CXCL8 and IL1B in cluster NO. 13 of CSF cells. One point represents one cell. Shades of red represent the relative expression (the higher the expression, the redder the color).

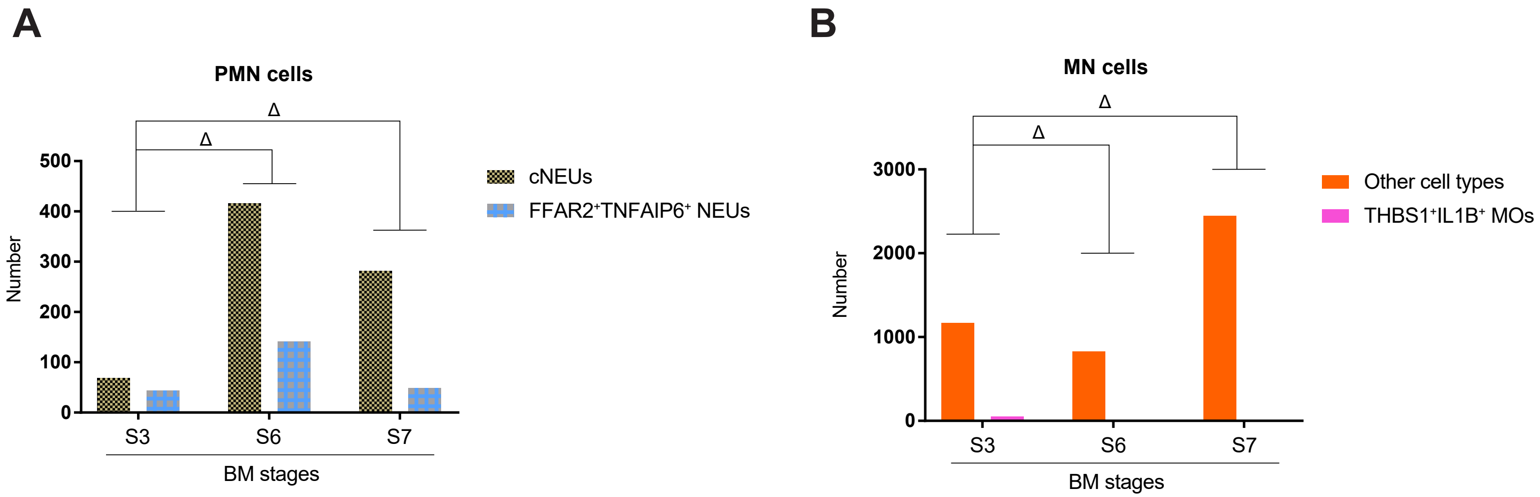

Figure S11. The higher frequencies of FFAR2$^+$TNFAIP6$^+$ NEUs and THBS1$^+$IL1B$^+$ MOs are identified by scRNA-seq in CSF cells of BM S3 stage than in S6 and S7 stages. (A) Grouped bar chart shows the numbers of cNEUs (yellow grid bars) and FFAR2$^+$TNFAIP6$^+$ NEUs (blue grid bars) in the BM S3, S6 and S7 stages. **(B)** Grouped bar chart shows the numbers of MNs excluding THBS1$^+$IL1B$^+$ MOs (orange bars) and THBS1$^+$IL1B$^+$ MOs (pink bars) in the BM S3, S6 and S7 stages. Δ: Chi-square test, $p < 0.05$.

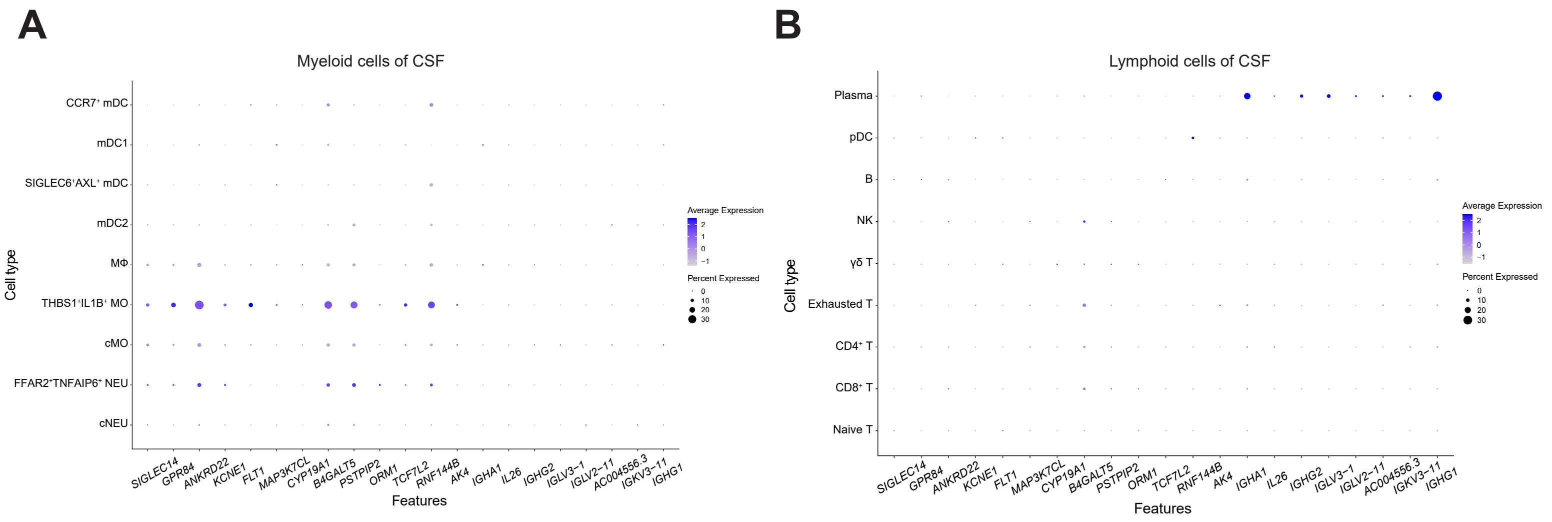

Figure S12. Bubble plots show the expression of gene set (*SIGLEC14, GPR84, ANKRD22, KCNE1, FLT1, MAP3K7CL, CYP19A1, B4GALT5, PSTPIP2, ORM1, TCF7L2, RNF144B, AK4, IGHA1, IL26, IGHG2, IGLV3-1, IGLV2-11, AC004556.3, IGKV3-11* and *IGHG1*) on myeloid cell types (A) and lymphoid cell types (B) of CSF. Shades of blue represent the relative abundance (the higher the abundance, the bluer the color), and bubble sizes represent the expression level (the higher the expression, the larger the size) of each gene.

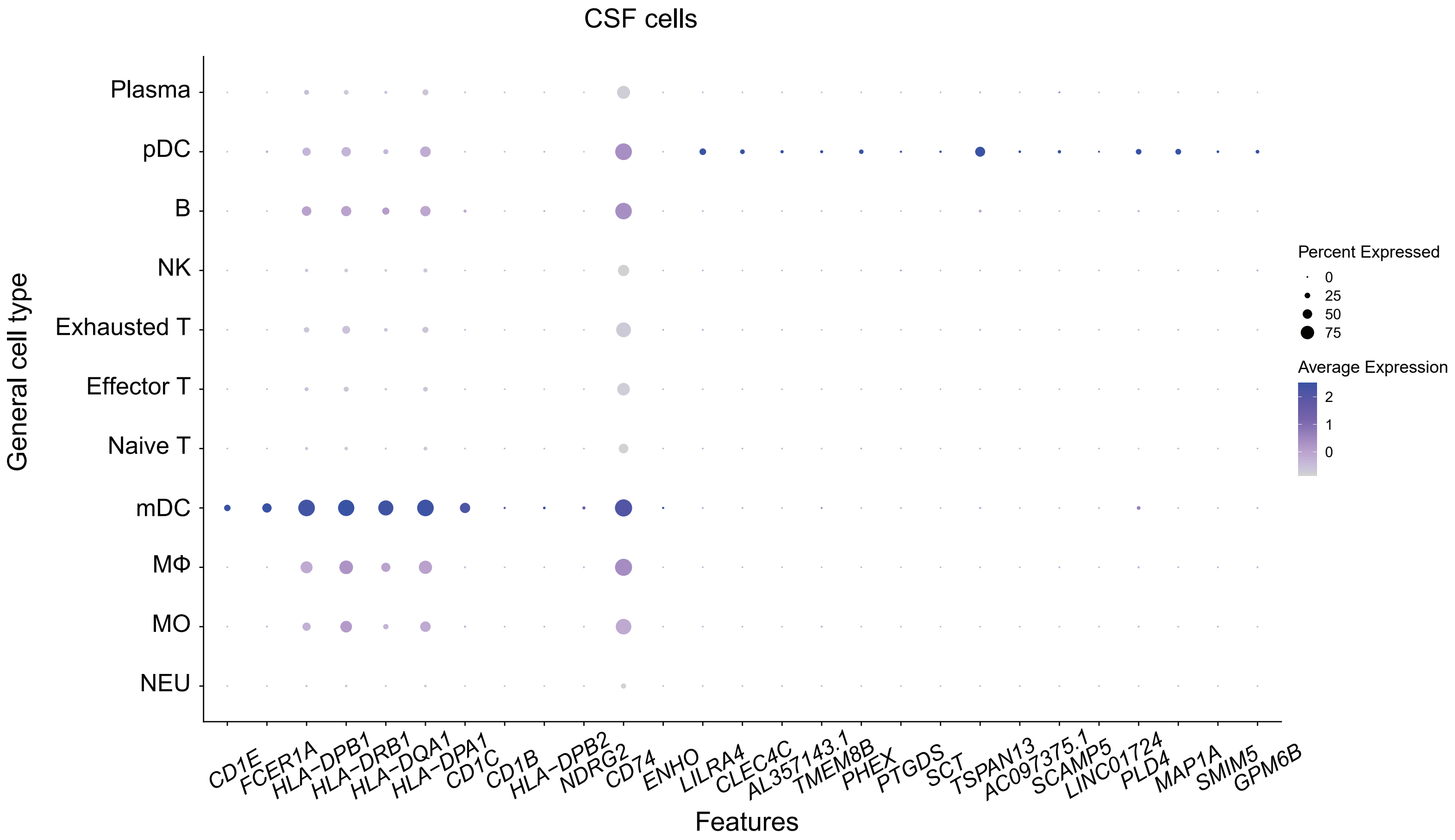

Figure S13. Bubble plot shows the expression of gene set (*CD1E, FCER1A, HLA−DPB1, HLA−DRB1, HLA−DQA1, HLA−DPA1, CD1C, CD1B, HLA−DPB2, NDRG2, CD74, ENHO, LILRA4, CLEC4C, AL357143.1, TMEM8B, PHEX, PTGDS, SCT, TSPAN13, AC097375.1, SCAMP5, LINC01724, PLD4, MAP1A, SMIM5* and *GPM6B*) in CSF general cell types. Shades of blue represent the relative abundance (the higher the abundance, the bluer the color), and bubble sizes represent the expression level (the higher the expression, the larger the size) of each gene.

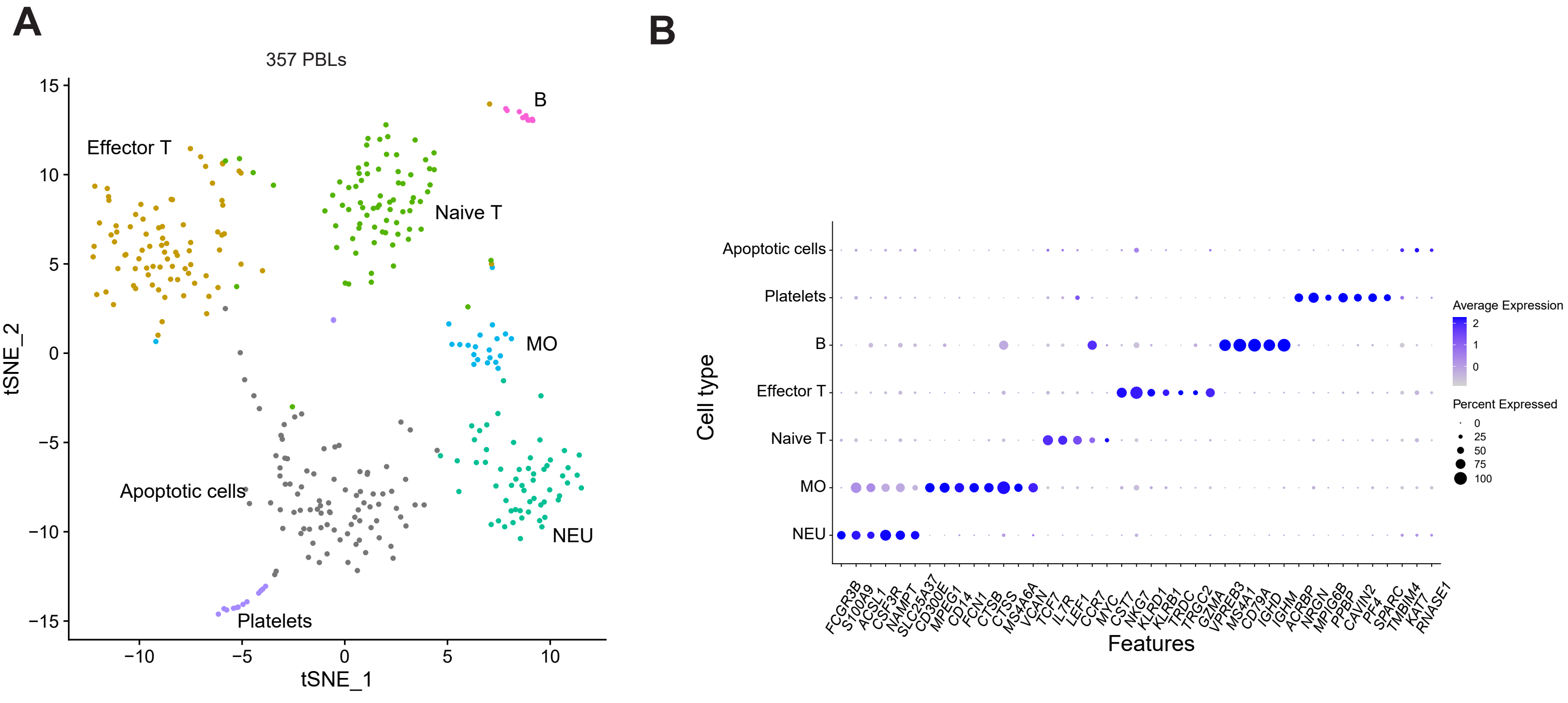

Figure S14. scRNA-seq reveals the cell heterogeneity of PBLs from sample B24. (A) The t-SNE plot shows the cell types of PBLs. One point represents one cell, and different cell clusters are marked by different colors. One cell cluster represents one cell type, and the name is marked on the side. **(B)** Bubble plot shows the feature genes of each cell type. Shades of blue represent the relative abundance (the higher the abundance, the bluer the color), and bubble sizes represent the expression level (the higher the expression, the larger the size) of each gene.

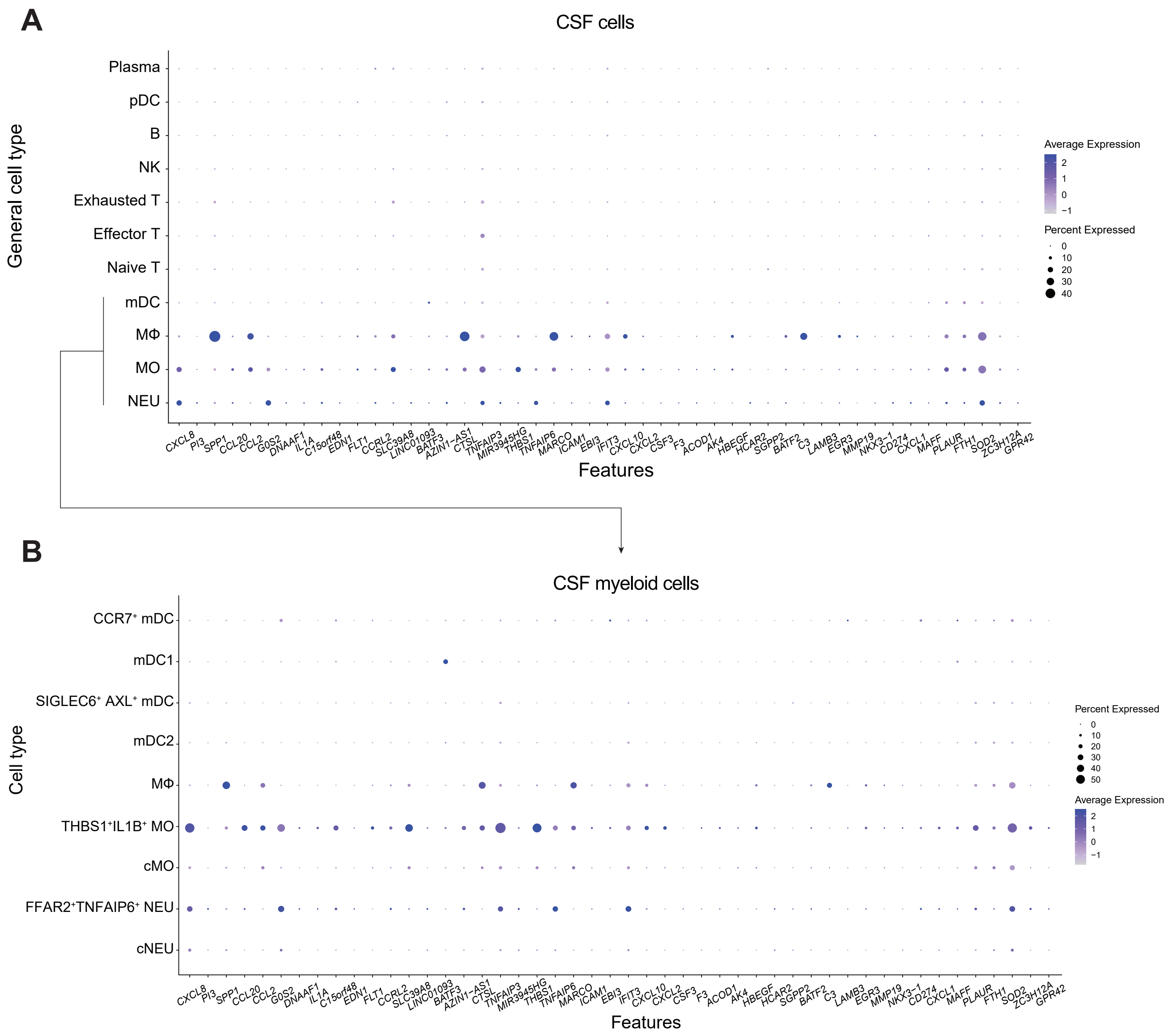

Figure S15. Bubble plots show the expression of gene set (*CXCL8, PI3, SPP1, CCL20, CCL2, G0S2, DNAAF1, IL1A, C15orf48, EDN1, FLT1, CCRL2, SLC39A8, LINC01093, BATF3, AZIN1-AS1, CTSL, TNFAIP3, MIR3945HG, THBS1, TNFAIP6, MARCO, ICAM1, EBI3, IFIT3, CXCL10, CXCL2, CSF3, F3, ACOD1, AK4, HBEGF, HCAR2, SGPP2, BATF2, C3, LAMB3, EGR3, MMP19, NKX3-1, CD274, CXCL1, MAFF, PLAUR, FTH1, SOD2, ZC3H12A* and *GPR42*) on CSF general cell types (A) and myeloid cell types (B). Shades of blue represent the relative abundance (the higher the abundance, the bluer the color), and bubble sizes represent the expression level (the higher the expression, the larger the size) of each gene.

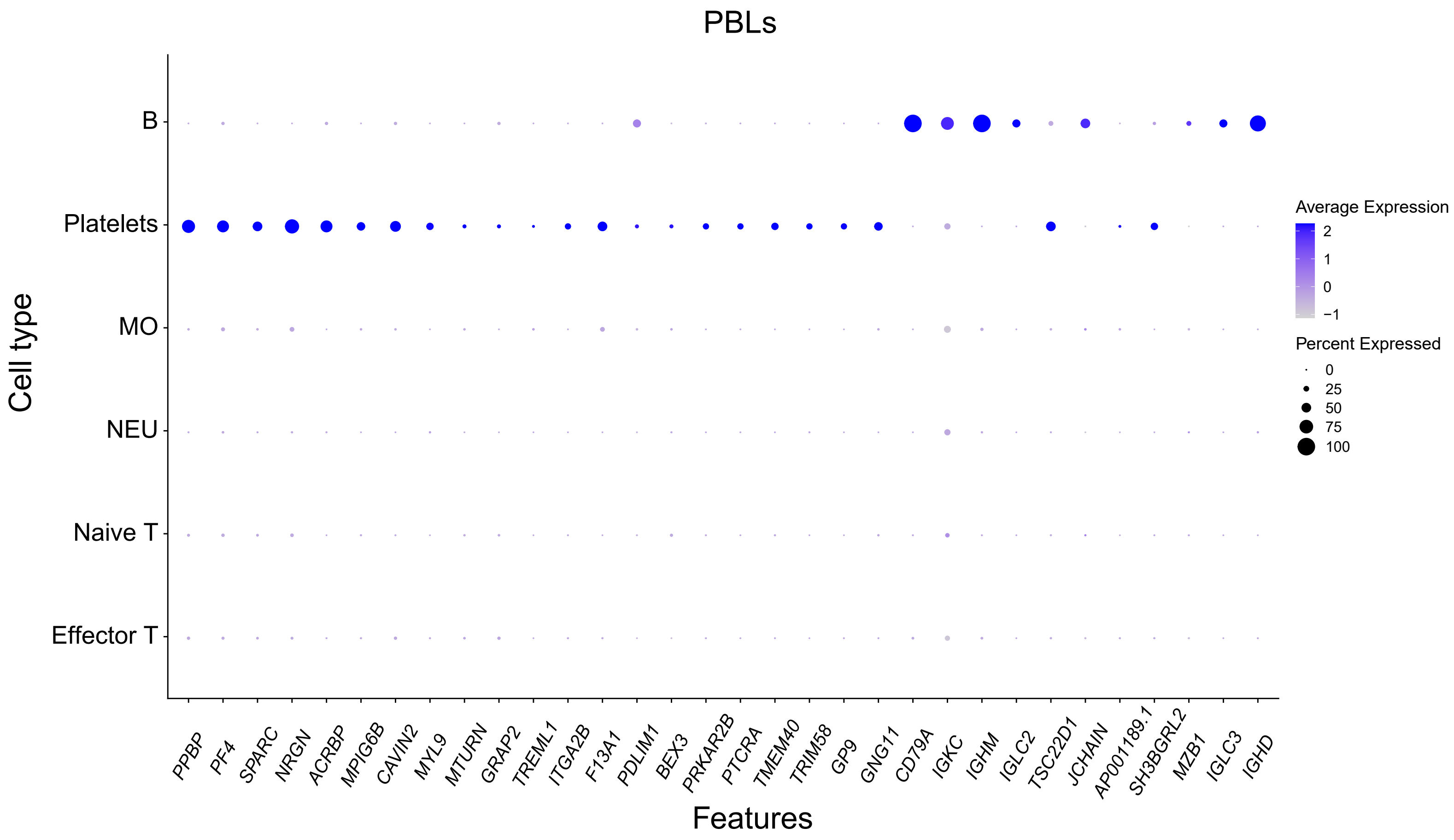

Figure S16. Bubble plot shows the expression of gene set (P*PBP, PF4, SPARC, NRGN, ACRBP, MPIG6B, CAVIN2, MYL9, MTURN, GRAP2, TREML1, ITGA2B, F13A1, PDLIM1, BEX3, PRKAR2B, PTCRA, TMEM40, TRIM58, GP9, GNG11, CD79A, IGKC, IGHM, IGLC2, TSC22D1, JCHAIN, AP001189.1, SH3BGRL2, MZB1, IGLC3* and *IGHD*) on cell types of PBLs from BM patients. Shades of blue represent the relative abundance (the higher the abundance, the bluer the color), and bubble sizes represent the expression level (the higher the expression, the larger the size) of each gene.

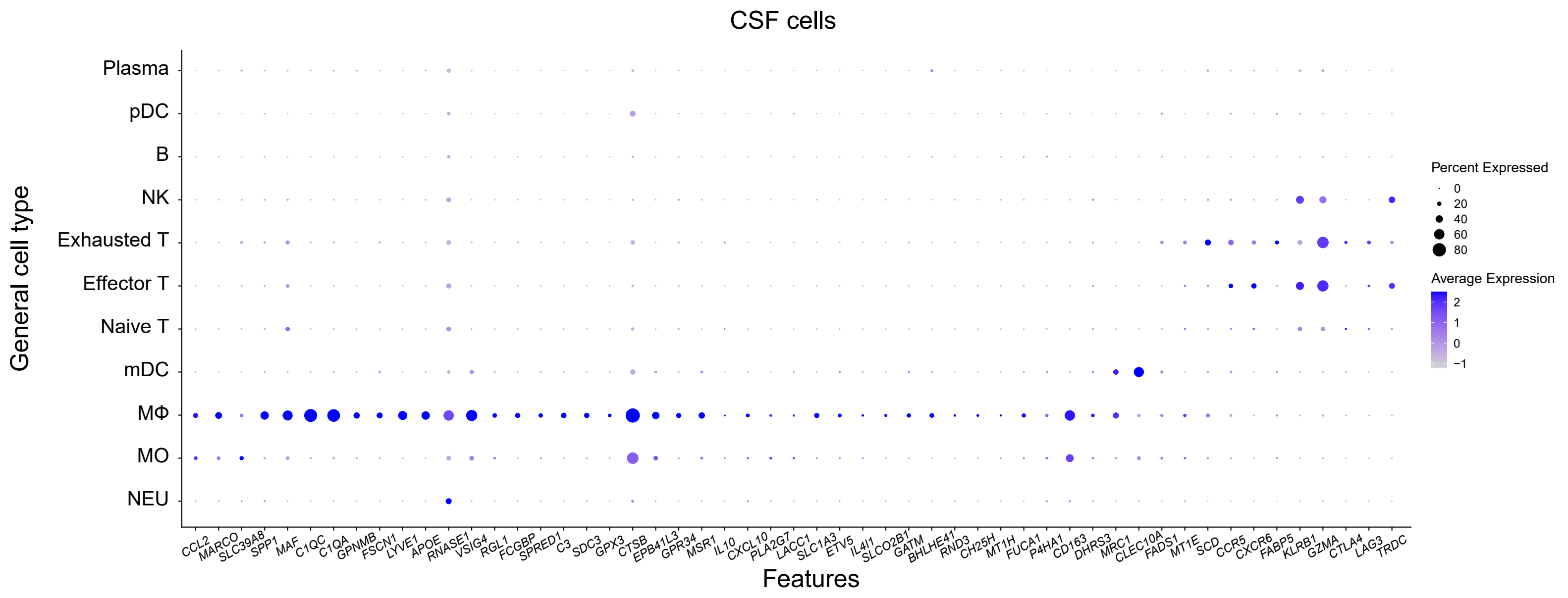

Figure S17. Bubble plot shows the expression of gene set (*CCL2, MARCO, SLC39A8, SPP1, MAF, C1QC, C1QA, GPNMB, FSCN1, LYVE1, APOE, RNASE1, VSIG4, RGL1, FCGBP, SPRED1, C3, SDC3, GPX3, CTSB, EPB41L3, GPR34, MSR1, IL10, CXCL10, PLA2G7, LACC1, SLC1A3, ETV5, IL4I1, SLCO2B1, GATM, BHLHE41, RND3, CH25H, MT1H, FUCA1, P4HA1, CD163, DHRS3, MRC1, CLEC10A, FADS1, MT1E, SCD, CCR5, CXCR6, FABP5, KLRB1, GZMA, CTLA4, LAG3* and *GPR42*) on CSF general cell types. Shades of blue represent the relative abundance (the higher the abundance, the bluer the color), and bubble sizes represent the expression level (the higher the expression, the larger the size) of each gene.

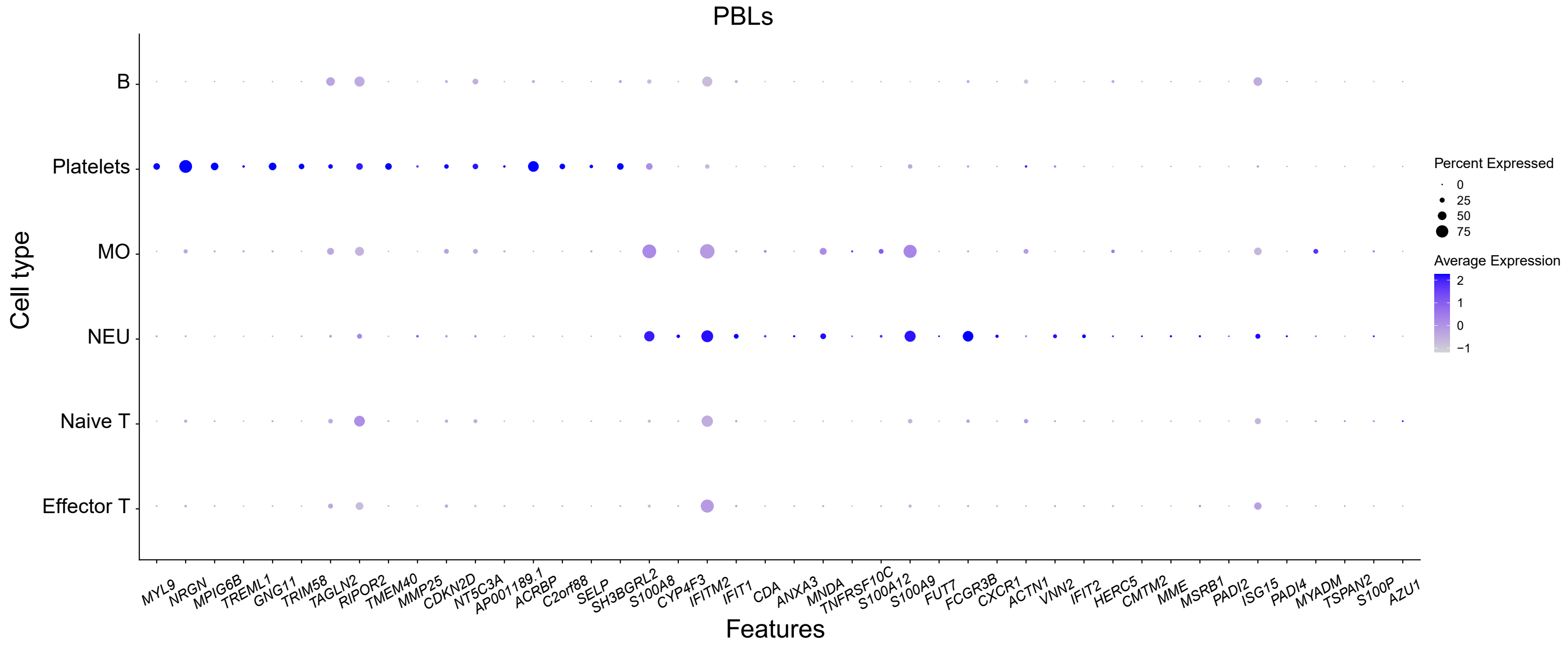

Figure S18. Bubble plot shows the expression of gene set (*MYL9, NRGN, MPIG6B, TREML1, GNG11, TRIM58, TAGLN2, RIPOR2, TMEM40, MMP25, CDKN2D, NT5C3A, AP001189.1, ACRBP, C2orf88, SELP, SH3BGRL2, S100A8, CYP4F3, IFITM2, IFIT1, CDA, ANXA3, MNDA, TNFRSF10C, S100A12, S100A9, FUT7, FCGR3B, CXCR1, ACTN1, VNN2, IFIT2, HERC5, CMTM2, MME, MSRB1, PADI2, ISG15, PADI4, MYADM, TSPAN2, S100P* and *AZU1*) on cell types of PBLs from BM patients. Shades of blue represent the relative abundance (the higher the abundance, the bluer the color), and bubble sizes represent the expression level (the higher the expression, the larger the size) of each gene.

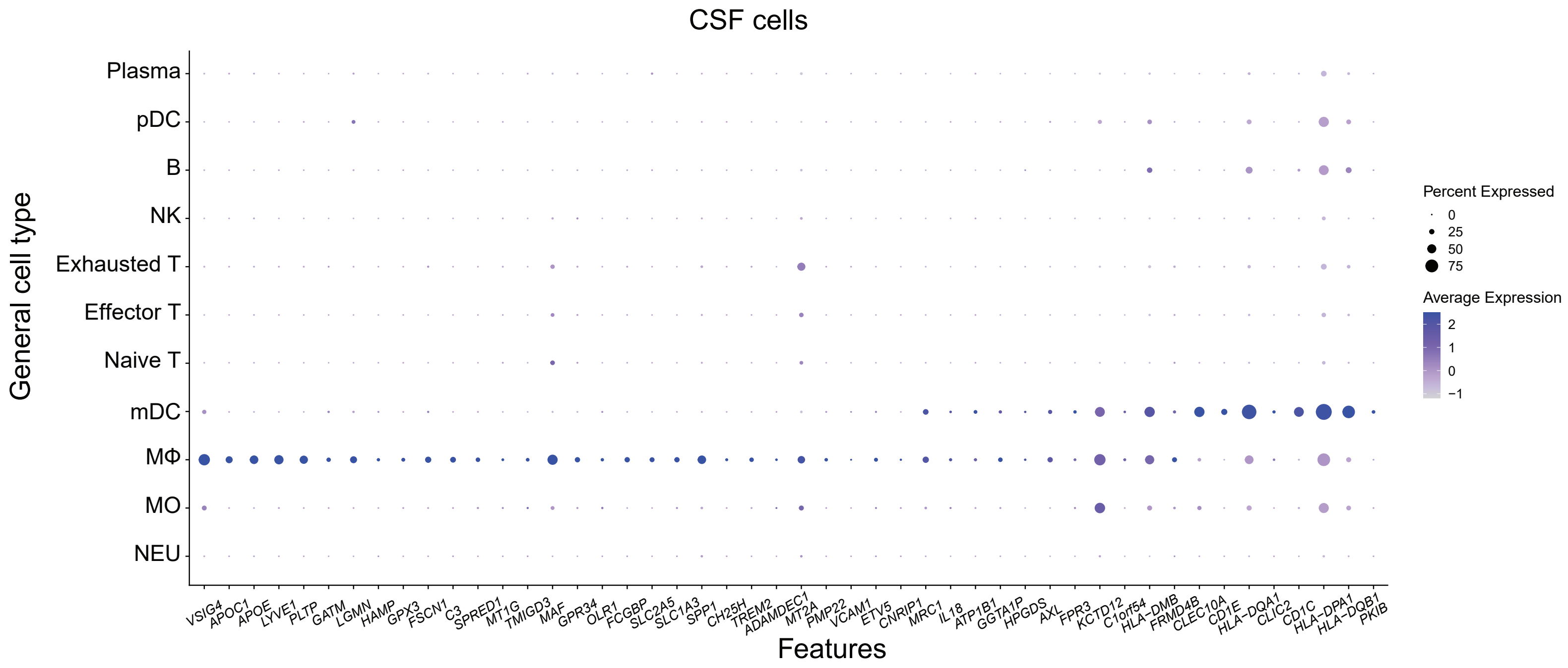

Figure S19. Bubble plot shows the expression of gene set (*VSIG4, APOC1, APOE, LYVE1, PLTP, GATM, LGMN, HAMP, GPX3, FSCN1, C3, SPRED1, MT1G, TMIGD3, MAF, GPR34, OLR1, FCGBP, SLC2A5, SLC1A3, SPP1, CH25H, TREM2, ADAMDEC1, MT2A, PMP22, VCAM1, ETV5, CNRIP1, MRC1, IL18, ATP1B1, GGTA1P, HPGDS, AXL, FPR3, KCTD12, C1orf54, HLA−DMB, FRMD4B, CLEC10A, CD1E, HLA−DQA1, CLIC2, CD1C, HLA−DPA1, HLA−DQB1* and *PKIB*) on CSF general cell types. Shades of blue represent the relative abundance (the higher the abundance, the bluer the color), and bubble sizes represent the expression level (the higher the expression, the larger the size) of each gene.

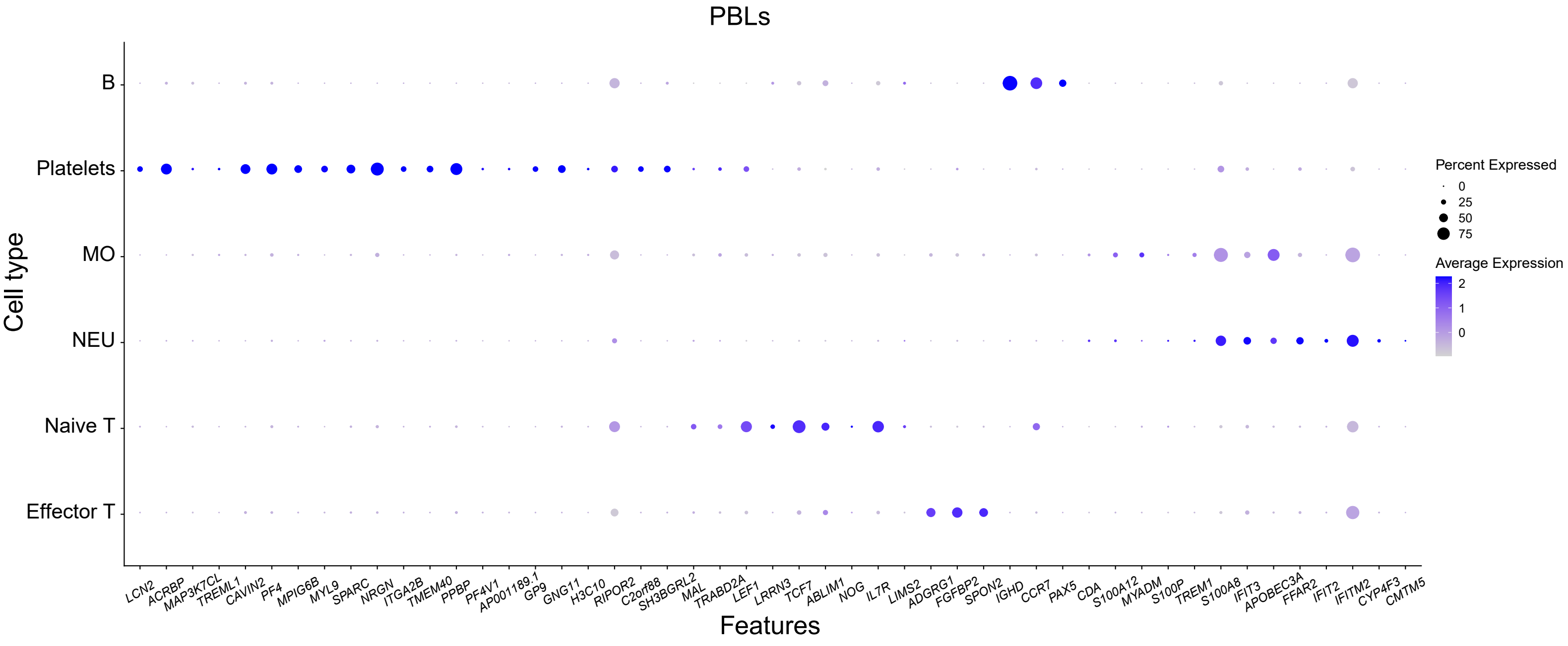

Figure S20. Bubble plot shows the expression of gene set (*LCN2, ACRBP, MAP3K7CL, TREML1, CAVIN2, PF4, MPIG6B, MYL9, SPARC, NRGN, ITGA2B, TMEM40, PPBP, PF4V1, AP001189.1, GP9, GNG11, H3C10, RIPOR2, C2orf88, SH3BGRL2, MAL, TRABD2A, LEF1, LRRN3, TCF7, ABLIM1, NOG, IL7R, LIMS2, ADGRG1, FGFBP2, SPON2, IGHD, CCR7, PAX5, CDA, S100A12, MYADM, S100P, TREM1, S100A8, IFIT3, APOBEC3A, FFAR2, IFIT2, IFITM2, CYP4F3* and *CMTM5*) on cell types of PBLs from BM patients. Shades of blue represent the relative abundance (the higher the abundance, the bluer the color), and bubble sizes represent the expression level (the higher the expression, the larger the size) of each gene.

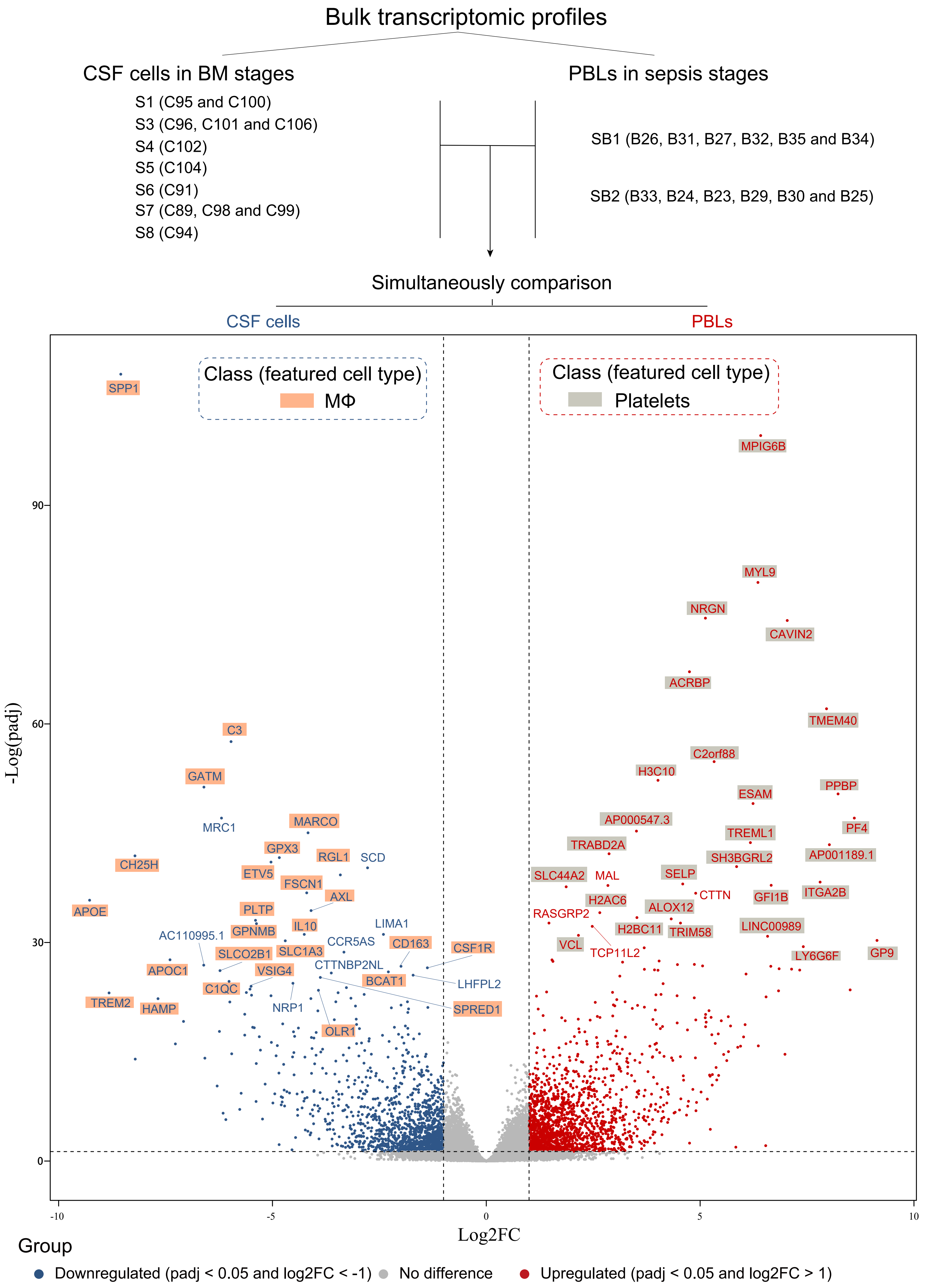

Figure S21. Volcano plot shows the DEGs of the simultaneous bulk transcriptomic comparison between CSF cells and PBLs in different onset stages of sepsis-developed BM. The x-axis shows log twofold change (log2FC) values, and the y-axis shows negative log ten padj values. Each gene is represented by a single spot. Significantly downregulated (padj < 0.05 and log2FC < -1) and upregulated (padj < 0.05 and log2FC > 1) DEGs are represented by blue and red spots, respectively. The thresholds of log2FC and padj are plotted with dashed lines. The names of downregulated and upregulated genes with top padj values are listed next to the corresponding spots. The background colors of the gene names represent the featured CSF cell types that highly express the corresponding genes.

Table legends

Table S1. Characteristics of CSF and blood samples collected from BM patients in the study.

Table S2. The sequences of the primers that are used in scRNA-seq and bulkRNA-seq.

Table S3. The sequences of the primer pairs that are used in RT-qPCR for human *TREM2*, *SLCO2B1* and *ACTB*.

Table S4. Ligand-receptor interactions in cell-cell communications of CSF cells that are identified with changed levels between in BM non-refractory and refractory remission stages.

Additional results

Additional result 1

293T cells were cultured in Dulbecco's modified Eagle's medium (DMEM) supplemented with 10% FBS, 2 mM L-glutamine (Gibco) and 100 units/mL penicillin-streptomycin (Thermo) at 37℃ and 5% CO2. Cells in logarithmic growth phase were collected by centrifugation at 600 ×g. An independent scRNA-seq targeting 293T cells was then performed, and the process was the same as that for CSF cells. We integrated the scRNA-seq data of 293T cells and CSF sample C57 for mixed clustering, and the results showed that CSF cells and 293T cells were clearly distinguished from each other. This verified the accuracy of scRNA-seq for CSF cells in our study.

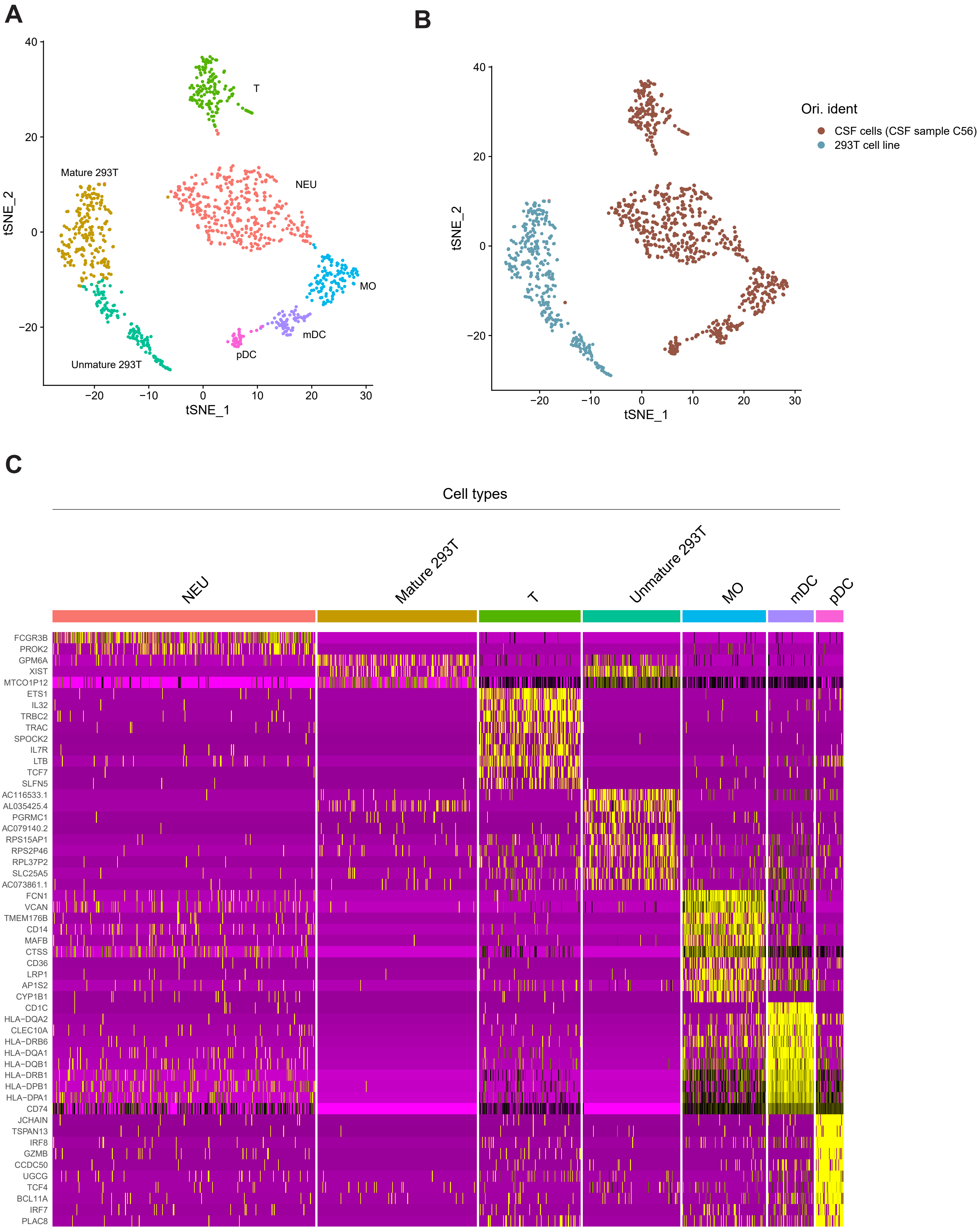

Figure. scRNA-seq reveals cell subsets in the mixed clustering of CSF cells and 293T cells. (**A**) t-SNE shows the cell subsets after mixed clustering of CSF cells and 293T cells. CSF cells are the scRNA-seq data of sample C56. (**B**) t-SNE shows the origin identity of each cell. (**C**) Heatmap shows the marker genes of each cluster. The black to yellow gradient represents low to high logFC of each gene.

Additional result 2

An independent scRNA-seq targeting CSF sample C138 (BM stage S5) was performed, and single-cell transcriptomes of 424 cells were obtained. Through clustering, these cells were identified as 228 naïve T cells, 153 effector T cells, 24 myeloid cells and 19 B cells. The results showed that T cells were the dominant cell type (90%) in the CSF. Since the patient was relatively cured at the BM S5 stage, this result was consistent with the composition of CSF cells in healthy individuals (Jenna L. Pappalardo, et al. Science Immunology, 2020).

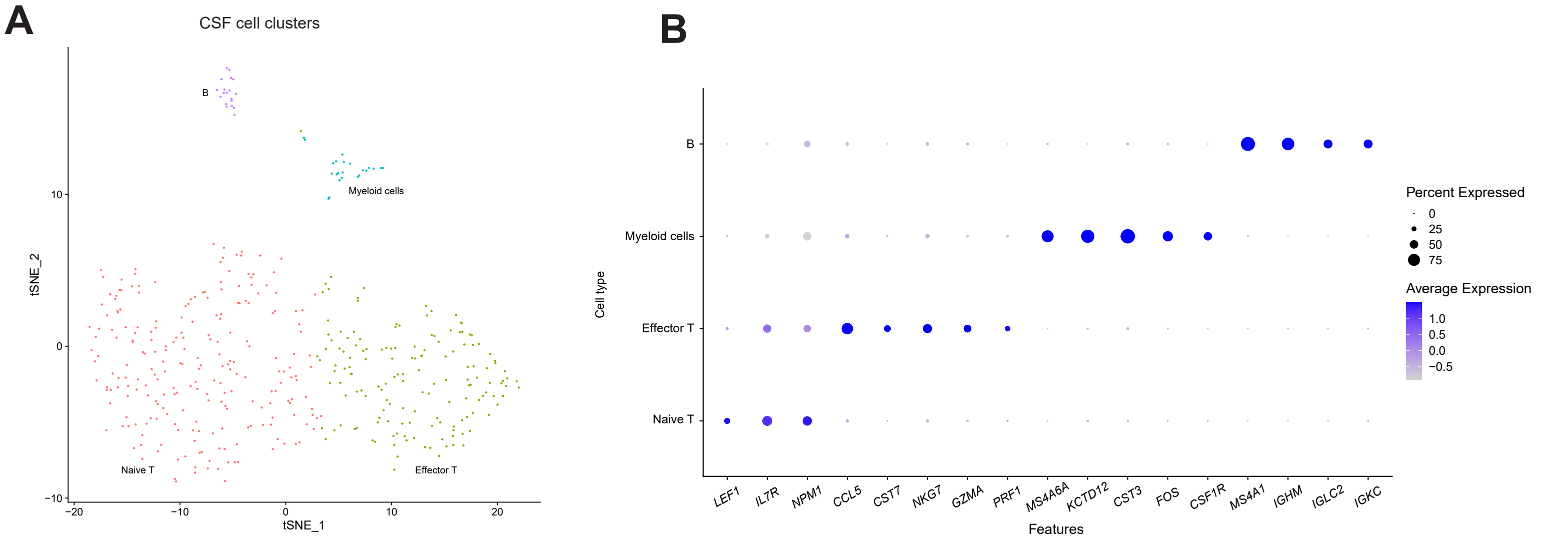

Figure. scRNA-seq reveals the cell subsets of CSF cells in the BM S5 stage. (**A**) t-SNE plot shows the cell subsets in CSF from sample C138. (**B**) Bubble plot shows the feature genes of each cluster. Shades of blue represent the relative abundance (the higher the abundance, the bluer the color), and bubble sizes represent the expression level (the higher the expression, the larger the size) of each gene.

Additional result 3

Bulk transcriptomic comparison between PBLs in the SB1 and SB2 conditions was performed. We found that down-regulated DEGs had GO enrichments in NEU-related immunity and immune responses to bacteria, while the up-regulated DEGs were enriched in T-cell activation and leukocyte cell-cell adhesion. The top down-regulated DEGs, such as LILRB2, LILRA6, FGR and ETS2, were mainly expressed in MOs and NEUs, and the top up-regulated DEGs, such as GNLY, KLRG1, TRDC, GZMA and SH2D1A, were mainly expressed in effector T cells. This revealed that the TCC increase in PBLs was accompanied by the growth of myeloid cells, especially NEUs, in sepsis occurrence, which was similar to the characteristic of CSF pleocytosis in BM.

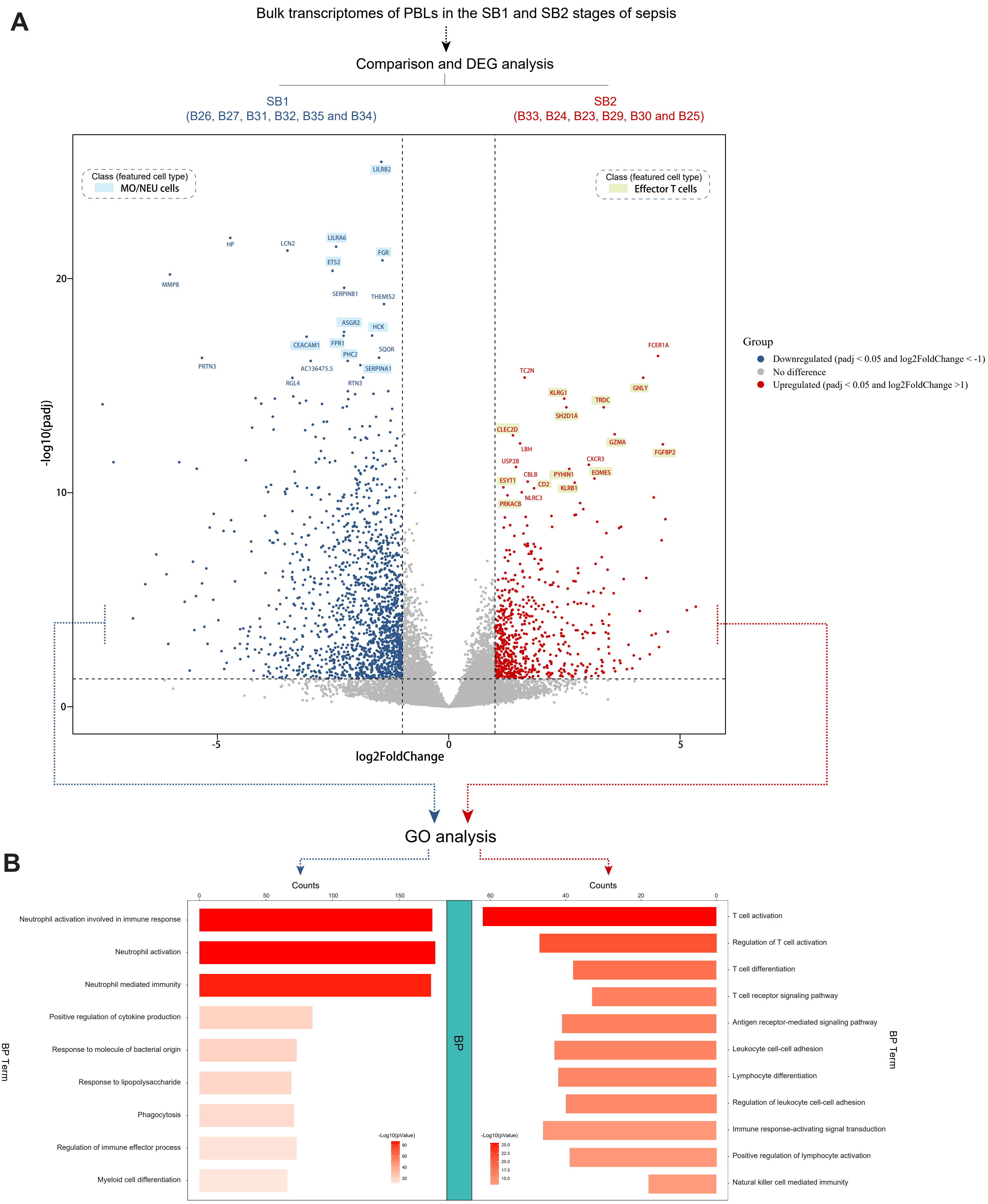

Figure. Different molecular hallmarks between PBLs in sepsis SB1 and SB2 stages. (**A**) Volcano plot shows the DEGs between PBLs in sepsis SB1 and SB2 stages. The x-axis shows log2FC values, and the y-axis shows negative log ten padj values. Each gene is represented by a single spot. Significantly downregulated (log2FC < -1 and padj < 0.05) and upregulated (log2FC > 1 and padj < 0.05) genes are represented by blue and red spots, respectively. The thresholds of log2FC and padj are plotted with dashed lines. The names of downregulated and upregulated genes with top padj values are listed next to the corresponding spots. The background colors of downregulated and upregulated gene names represent the cell types of CSF and PBLs, respectively, which highly express the corresponding gene. (**B**) Bar charts show the terms of GO-BP enrichments targeting the downregulated and upregulated DEGs in the comparison. shades of red indicate the log P value of each GO-BP term from low to high (the more significant the P value is, the redder the color) (scaled). Bar lengths indicate the number of genes enriched for each GO-BP term.
