# Supplementary material for: High-throughput sequencing unravels the cell heterogeneity of cerebrospinal fluid in the bacterial meningitis of children
Source: Front Immunol. 2022 Sep 2;13:872832. doi: 10.3389/fimmu.2022.872832 (PMC9478118; doi:10.3389/fimmu.2022.872832)
Supplement: Supplementary file 2 [file Table_1.pdf]

Table S1. Characteristics of CSF and blood samples collected from BM patients in the study.

Patient.NO.	Gender	Age/Days	Pathogen	CSF.NO.	Onset Duration of sampling CSF/Days	TCC of CSF / ×10^6/L	PMN cells /×10^6/L	MN cells /×10^6/L	CSF Test	BM stage	Blood.NO.	Onset Duration of sampling blood/Days	TCC of PBLs / ×10^9/L	Blood Test	Sepsis stage
1	Male	48	*Streptococcus agalactiae*	C1	75	16	2	14	1-1, 2-1	S7	B1	71	8.79	1-2, 2-2	SB2
2	Male	41	Unclear	C2	85	40	4	36	1-1	S7	B2	94	3.77	1-2, 2-2	SB1
3	Female	67	*Streptococcus agalactiae*	C3	13	52	16	36	1-1, 2-1	S3	B3	14	16.24	1-2, 2-2	SB1
4	Male	25	*Streptococcus agalactiae*	C4	39	42	13	29	1-1	S7	B4	40	7.84	1-2, 2-2	SB2
5	Male	5	*Streptococcus agalactiae*	C5	37	38	10	28	1-1, 2-1	S7	B5	43	10.89	1-2, 2-2	SB1
6	Male	53	*Streptococcus agalactiae*	C6	30	21	6	15	1-1, 2-1	S7	B6	39	4.30	1-2, 2-2	SB2
7	Female	75	*Escherichia coli*	C7	8	0	0	0		S5	B7	34	6.05		SB2
				C8	34	10	0	10	2-1	S8					
8	Female	2	*Streptococcus agalactiae*	C9	62	16	0	16	1-1	S8	B8	64	10.38	1-2, 2-2	SB1
9	Male	70	*Streptococcus agalactiae*	C10	42	6	0	6	2-1	S8	B9	45	5.55	2-2	SB2
10	Female	129	*Streptococcus pneumonia*	C11	9	20	1	19	2-1	S3	B10	13	6.13		SB2
				C12	21	10	0	10		S4					
11	Male	67	*Streptococcus agalactiae*	C13	42	76	49	27		S6	B11	40	5.15		SB2
12	Female	600	*Streptococcus pneumonia*	C14	36	20	5	15	2-1	S7	B12	37	6.14	2-2, 3-2	SB2
13	Female	236	*Streptococcus pneumonia*	C15	1	1000	900	100		S1	B13	3	18.74	2-2	SB1
				C16	4	256	225	31	2-1	S1					
				C17	9	60	25	35		S3	B14	10	10.20		SB1
				C18	16	5	0	5	6	S4					
14	Male	299	Unclear	C19	12	6	0	6	2-1	S4	B15	13	12.35	2-2	SB1
15	Female	8	*Escherichia coli*	C20	80	10	0	10		S8	B16	75	4.33	3-2	SB2
16	Male	27	*Escherichia coli*	C21	2	5000	3000	2000		S1	B17	30	8.26	3-2	SB2
				C22	4	5180	4144	1036		S1					
				C23	9	61	1	60		S3					
				C24	17	30	3	27	2-3, 3-3, 3-4	S3					
				C25	22	80	8	72		S7					
				C26	29	60	20	40		S7					
				C27	36	54	9	45		S7					
				C28	42	40	10	30		S7					
				C29	57	12	0	12		S8					
				C30	71	3	0	3		S8					
				C31	86	18	6	12		S7					
				C32	99	15	0	15		S8					
				C33	107	0	0	0		S9					
				C34	113	6	0	6		S8					
17	Male	154	Unclear	C35	11	4	0	4		S4	B18	14	3.88	3-2, 6	SB1
18	Male	476	*Streptococcus pneumonia*	C36	23	0	0	0		S5	B19	25	4.00	3-2, 2-2	SB2
19	Female	315	*Streptococcus pneumonia*	C37	49	10	0	10		S8	B20	49	11.08	3-2	SB1
20	Male	143	*Streptococcus pneumonia*	C38	11	154	69	85		S2	B21	17	4.52	3-2	SB2
				C39	18	15	0	15		S4					
				C40	26	39	3	36		S7					
				C41	30	8	0	8		S8					
				C42	37	7	0	7		S8					
				C43	43	10	0	10		S8					
21	Female	47	Unclear	C44	1	800	480	320		S1	B22	4	26.76	3-2, 2-2	SB1
				C45	4	0	0	0		S5					
22	Male	384	*Streptococcus pneumonia*	C46	80	0	0	0	6	S9					
23	Male	88	*Escherichia coli*	C47	49	146	88	58	2-3, 3-3, 3-4, 4	S6					
24	Male	61	Unclear	C48	56	12	0	12		S8					
				C49	63	2	0	2		S8					
25	Male	31	*Escherichia coli*	C50	9	0	0	0		S5					
				C51	16	0	0	0		S5					
26	Female	137	*Streptococcus pneumonia*	C52	14	0	0	0		S5					
27	Male	3	*Escherichia coli*	C53	72	2	0	2	6	S8					
28	Male	9	*Enterococcus Faecium*	C54	29	102	80	22		S6					
				C55	32	126	44	82	3-4, 4, 6	S7					
				C56	39	220	114	106	3-1, 3-3, 3-4, 4, 6	S6					
				C57	44	78	26	52	3-3, 3-4, 4	S7					
				C58	54	104	62	42	3-4, 4	S6					
				C59	63	75	45	30	3-4, 4	S6					
				C60	77	26	10	16	3-4	S7					
				C61	87	24	4	20	3-1, 3-4, 4	S7					
				C62	102	10	0	10	3-1, 3-4, 4	S8					
29	Male	768	*Streptococcus pneumonia*	C63	3	930	744	186		S1					
				C64	8	160	90	70	3-1， 3-4, 6	S2					
				C65	15	49	2	47	4	S3					
				C66	22	35	12	23	4	S3					
				C67	29	42	10	32	4	S7					
				C68	36	28	12	16		S7					
				C69	50	5	0	5	3-4	S8					
30	Male	176	*Streptococcus pneumonia*	C70	11	80	12	68		S3					
				C71	18	20	8	12	3-3, 3-4, 4	S7					
				C72	25	22	6	16	3-3, 3-4, 4	S7					
				C73	32	14	0	14	4	S8					
31	Male	137	*Escherichia coli*	C74	2	7618	5691	1927		S1					
				C75	5	42	4	38		S3					
				C76	13	20	5	15	2-3, 3-3， 3-4	S3					
				C77	18	9	0	9	3-4, 4	S4					
				C78	30	30	4	26		S7					
				C79	35	28	6	22		S7					
				C80	42	8	0	8	3-1, 3-4, 4	S8					
				C81	49	2	0	2	3-4	S8					
32	Male	55	*Streptococcus agalactiae*	C82	18	4	0	4		S4					
33	Male	96	*Neisseria meningitidis*	C83	15	169	88	81		S2					
				C84	23	48	5	43	2-3	S3					
				C85	30	191	29	162		S7					
				C86	37	167	32	135		S7					
34	Female	95	*Streptococcus agalactiae*	C87	7	2	0	2		S4	B23	21	9.69	5-2, 2-2	SB2
				C88	14	30	10	20		S7					
				C89	21	24	10	14	3-3, 3-4, 5-1	S7					
				C90	27	2	0	2		S8					
35	Female	1	*Streptococcus agalactiae*	C91	67	58	38	20	3-3, 3-4, 5-1	S6	B24	66	7.68	5-2, 4	SB2
				C92	72	24	10	14		S7					
				C93	77	6	0	6		S8					
36	Male	65	*Streptococcus agalactiae*	C94	29	12	0	12	3-4, 5-1	S8	B25	29	5.76	5-2	SB2
37	Male	56	*Escherichia coli*	C95	3	130	111	19	3-3, 3-4, 5-1	S1	B26	3	1.79	5-2	SB1
				C96	12	23	4	19	3-1,3-3, 3-4, 5-1	S3	B27	11	21.72	5-2	SB1
				C97	27	120	12	108		S7	B28	18	5.7		SB2
				C98	32	24	4	20	3-3, 3-4, 5-1	S7	B29	34	4.62	5-2	SB2
				C99	46	41	3	38	3-1, 3-3, 3-4, 5-1	S7	B30	44	4.15	5-2	SB2
38	Male	38	*Streptococcus agalactiae*	C100	1	10300	8446	1854	3-1, 3-4, 4, 5-1	S1	B31	3	12.43	5-2	SB1
				C101	7	40	16	24	4, 5-1	S3	B32	7	10.82	5-2	SB1
				C102	14	4	0	4	3-4, 4, 5-1	S4	B33	14	5.94	5-2	SB2
39	Male	277	*Streptococcus pneumonia*	C103	3	1226	1030	196	4	S1	B34	10	11.89	5-2	SB1
				C104	10	0	0	0	3-4, 5-1	S5					
40	Female	29	*Streptococcus agalactiae*	C105	1	1978	1622	356		S1	B35	7	13.07	5-2	SB1
				C106	5	40	14	26	3-3， 3-4, 4, 5-1	S3					
				C107	12	26	6	20	4	S3					
				C108	19	31	3	28		S7					
				C109	27	39	5	34		S7					
				C110	57	56	15	41		S7					
				C111	64	50	10	40		S7					
				C112	78	17	2	15		S7					
41	Male	80	*Streptococcus agalactiae*	C113	45	187	150	37	2-3, 3-1, 3-3, 3-4	S6					
				C114	52	35	9	26	2-3, 3-3, 3-4	S7					
				C115	59	125	40	85	2-3, 3-3, 3-4	S7					
42	Male	48	Unclear	C116	11	6	0	6	3-4	S4					
				C117	18	7	0	7	3-4	S4					
43	Male	53	*Streptococcus agalactiae*	C118	13	31	5	26	2-3, 3-3, 3-4	S3	B36	20	12.33	3-2	SB1
				C119	20	12	0	12	3-1, 3-4, 4	S4					
44	Male	27	*Escherichia coli*	C120	78	9	1	8	3-1, 3-4	S8					
45	Female	39	*Streptococcus agalactiae*	C121	1	2210	1989	221	3-1, 3-4, 4	S1	B37	3	9.87	3-2	SB2
				C122	7	53	3	50	2-3, 3-1, 3-3, 3-4, 4	S3					
				C123	13	46	6	40	6	S3					
				C124	20	10	0	10		S4					
				C125	24	0	0	0	3-4, 6	S5					
46	Female	292	*Escherichia coli*	C126	10	87	29	58	2-3, 3-1,3-3， 3-4, 4	S3	B38	14	12.37	3-2	SB1
				C127	14	0	0	0	3-4	S5					
				C128	20	10	0	10	3-4	S4					
47	Female	1201	*Listeria monocytogenes*	C129	47	2	0	2	3-4, 4	S8	B39	40	2.9	3-2	SB2
				C130	54	20	7	13	2-3, 3-4	S7					
48	Male	62	*Streptococcus agalactiae*	C131	3	2832	2492	340	3-3, 3-4, 4, 6	S1					
				C132	10	4	0	4	3-4	S4					
				C133	16	0	0	0	3-4	S5					
49	Female	15	*Streptococcus agalactiae*	C134	10	230	161	69	2-3, 3-3	S2					
				C135	17	41	22	19	2-3	S2					
50	Male	897	*Listeria monocytogenes*	C136	37	0	0	0	3-4	S9					
51	Male	2490	*Streptococcus pneumonia*	C137	12	37	15	22	3-3， 3-4	S3					
				C138	17	0	0	0	4	S5					
52	Male	330	*Streptococcus pneumonia*	C139	6	8	0	8	3-4, 4	S4					
53	Male	88	*Escherichia coli*	C140	1	1300	1013	320	3-4	S1					
54	Male	143	*Escherichia coli*	C141	2	695	341	354	3-4	S1					

Note

1-1	RT-qPCR of CSF cells for *TREM2* , *SLCO2B1* and *β-actin*
1-2	RT-qPCR of PBLs for *TREM2* , *SLCO2B1* and *β-actin*
2-1	Immunostaining of CSF cells for VSIG4
2-2	Immunostaining of PBLs for VSIG4
2-3	Immunostaining of CSF cells for CD1C
3-1	Elisa of CSF supernatant for OPN (*SPP1*)
3-2	Elisa of blood plasma for OPN (*SPP1*)
3-3	Elisa of CSF supernatant for PEDF (*SERPINF1*)
3-4	Elisa of CSF supernatant for CXCL8
4	scRNA-seq of CSF cells
5-1	bulkRNA-seq of CSF cells
5-2	bulkRNA-seq of PBLs
6	Monitoring of PMNs and MNs populations by FCM
