# Supplementary material for: High-throughput sequencing unravels the cell heterogeneity of cerebrospinal fluid in the bacterial meningitis of children
Source: Front Immunol. 2022 Sep 2;13:872832. doi: 10.3389/fimmu.2022.872832 (PMC9478118; doi:10.3389/fimmu.2022.872832)
Supplement: Supplementary file 3 [file Table_2.pdf]

Table S2. The sequences of the primers that are used in scRNA-seq and bulkRNA-seq.

Name	Sequence
X908	5'-CAAGCAGAAGACGGCATACGAGAT-3'
X126	5'-ACATTGTATAGAATTCGCGGCCGCTCGCGATAC-3'
X899	5'-GTGACTGGAGTTCAGACGTGTGCTCTTCCGATCT-3'
X910	5'-TATAGAATTCGCGGCCGCTCGCGATACATCAATCNNNNNNNNNNNNNNNNTTTTTTTTTTTTTTTTTTTTTTTT-3'
