# Supplementary material for: High-throughput sequencing unravels the cell heterogeneity of cerebrospinal fluid in the bacterial meningitis of children
Source: Front Immunol. 2022 Sep 2;13:872832. doi: 10.3389/fimmu.2022.872832 (PMC9478118; doi:10.3389/fimmu.2022.872832)
Supplement: Supplementary file 4 [file Table_3.pdf]

Table S3. The sequences of the primer pairs that are used in RT-qPCR for human *TREM2* , *SLCO2B1* and *ACTB* .

Name	Sequence
TREM2	Forward: 5'-GCACAGCCATCACAGACGAT-3'<br>Reverse: 5'-CTCTGGCACTGGTAGAGACC-3'
SLCO2B1	Forward: 5'-GAAGCCAGAGGATTCCCGAG-3'<br>Reverse: 5'-CAGGGCTCTTGACACCCAAT-3'
ACTB	Forward: 5'-CAAAGTTCACAATGTGGCCGAG-3'<br>Reverse: 5'-GGACTGGGCCATTCTCCTTAG-3'
