# Supplementary material for: High-throughput sequencing unravels the cell heterogeneity of cerebrospinal fluid in the bacterial meningitis of children
Source: Front Immunol. 2022 Sep 2;13:872832. doi: 10.3389/fimmu.2022.872832 (PMC9478118; doi:10.3389/fimmu.2022.872832)
Supplement: Supplementary file 5 [file Table_4.pdf]

Table S4. Ligand-receptor interactions in cell-cell communications of CSF cells that are identified with changed levels between in BM non-refractory and refractory remission stages.

Cytokine-receptors	Cell adhesion	Immune response	Immune suppression	Immune checkpoints
CCL2_CCR2	CD1D_LILRB2	ADGRG5_FAM3C	ANXA1_FPR1	LGALS9_CD44
CCL22_DPP4	CD2_CD58	ADORA3_ENTPD1	ANXA1_FPR2	LGALS9_CD47
CCL3L1_CCR1	CD226_NECTIN2	ALOX5_ALOX5AP	ANXA1_FPR3	LGALS9_HAVCR2
CCL3L1_DPP4	CD47_SIRB1 complex	C3_aMb2 complex	APP_FPR2	LGALS9_COLEC12
CCL4_CCR5	CD47_SIRPG	C3_C3AR1	AXL_GAS6	LGALS9_LRP1
CCL4_SLC7A1	CD55_ADGRE5	CD74_COPA	BTLA_TNFRSF14	LGALS9_SLC1A5
CCL4L2_VSIR	CD6_ALCAM	COPA_SORT1	C5AR1_RPS19	LGALS9_SORL1
CCL5_CCR1	HGF_CD44	CXCR1_YARS	CD52_SIGLEC10	CTLA4_CD86
CCL5_CCR5	LAIR1_LILRB4	FAM3C_CLEC2D	EFNA2_EPHA4	CD28_CD86
CXCL10_CXCR3	PECAM1_CD38	FFAR2_FAM3C	KLRB1_CLEC2D	
CXCL10_DPP4	PLAUR_a4b1 complex	GRN_SORT1	LILRA4_BST2	
CXCL12_CXCR3	PLXNB2_SEMA4D	LAMP1_FAM3C	MERTK_GAS6	
CXCL12_CXCR4	PTPRC_MRC1	NRG1_MS4A4A	PTPRC_CD22	
CXCL8_CXCR1	SELL_SELPLG	NRP1_VEGFB	SIRPA_CD47	
CXCL8_CXCR2	SEMA4A_PLXND1	SPP1_PTGER4	TNFRSF1A_GRN	
CXCR3_CXCL9	SPN_SIGLEC1		TNFRSF1B_GRN	
CXCR6_CXCL16	SPP1_a4b1 complex			
DPP4_CXCL12	SPP1_CD44			
DPP4_CXCL9				
FCGR2A_CXCL9				
FFAR2_CCL4L2				
LTBR_LTB				
TNFRSF13C_TNFSF13B				
TNFRSF17_TNFSF13B				
TNFSF14_LTBR				
TNFSF14_TNFRSF14				
TFRC_TNFSF13B				
TNFSF10_RIPK1				
TGFB1_TGFBR1				
TGFB1_TGFBR3				
XCR1_XCL2				
